# Supplementary material for: Lighting up metal nanoclusters by the H2O-dictated electron relaxation dynamics
Source: Nat Commun. 2025 Mar 7;16:2295. doi: 10.1038/s41467-025-57554-7 (PMC11889173; doi:10.1038/s41467-025-57554-7)
Supplement: Supplementary file 1 — Supplementary Information [file 41467_2025_57554_MOESM1_ESM.pdf]

Supplementary Information

Lighting up metal nanoclusters by the H_2O-dictated electron relaxation dynamics

Yuan Zhong,[1,†] Xue Wang,[1,†] Zhou Huang,[2,†] Yao Wei,[3,†] Qing Tang,[2] Songqi Gu,[3*] Tingting Li,[4] Weinan Dong,[1] Feng Jiang,[1] Haifeng Zhu,[1] Yujia Shi,[5] Zhi Zhou,[6] Yu Zhang,[1*] Xue Bai,[1*] Zhennan Wu[1*]*

[1]State Key Laboratory of Integrated Optoelectronics, College of Electronic Science and Engineering, Jilin University, Changchun 130012, P. R. China

[2]Chongqing Key Laboratory of Chemical Theory and Mechanism, School of Chemistry and Chemical Engineering, Chongqing University, Chongqing 401331, P. R. China

[3]Shanghai Advanced Research Institute, Chinese Academy of Sciences, Shanghai 201210, P. R. China

[4]College of Materials Science and Engineering, Jilin Jianzhu University, Changchun 130012, P. R. China

[5]Department of Oral Implantology, Jilin Provincial Key Laboratory of Sciences and Technology for Stomatology Nanoengineering, School and Hospital of Stomatology, Jilin University, Changchun 130012, P. R. China

[6]Hunan Optical Agriculture Engineering Technology Research Center, School of Chemistry and Materials Science, Hunan Agricultural University, Changsha 410128, P. R. China

[†]These authors contributed equally: Yuan Zhong, Xue Wang, Zhou Huang, Yao Wei.

*Corresponding author. E-mail: qingtang@cqu.edu.cn; gusq@sari.ac.cn; yuzhang@jlu.edu.cn; baix@jlu.edu.cn; wuzn@jlu.edu.cn

Supplementary Figures

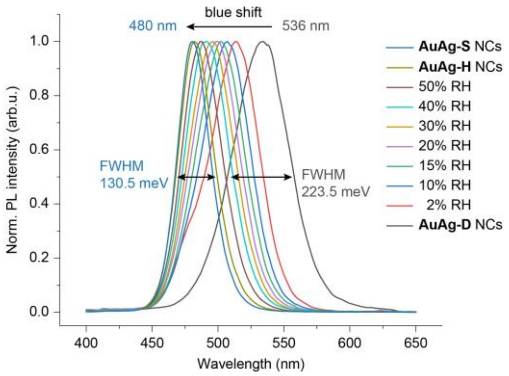

Supplementary Fig. 1. Normalized PL spectra of AuAg-D, AuAg-H, and AuAg-S NCs upon 365 nm excitation. The humidity condition for aging AuAg-D NCs is set from 2% to 56% RH.

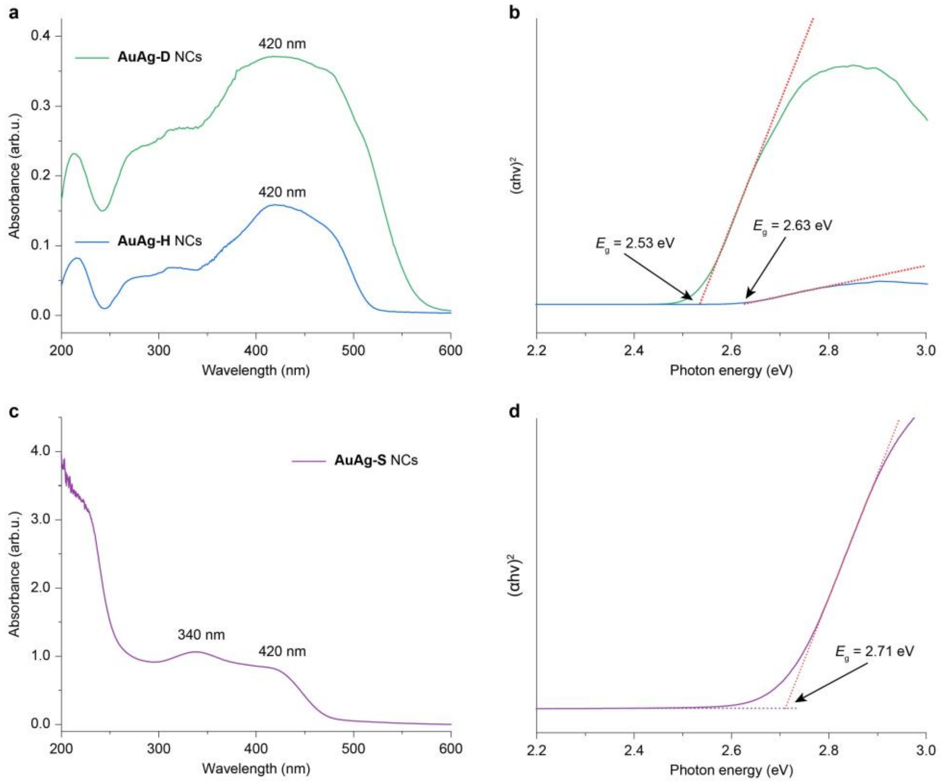

Supplementary Fig. 2. Absorption spectra and band gap calculations. a Solid-state UV-vis absorption spectra of AuAg-D and AuAg-H NCs. **b** Bandgap energy calculations of AuAg-D and AuAg-H NCs through the Tauc analyses. **c** UV-vis absorption spectra of the aqueous solution of AuAg-S NCs. **d** Bandgap energy calculation of AuAg-S NCs through the Tauc analyses.

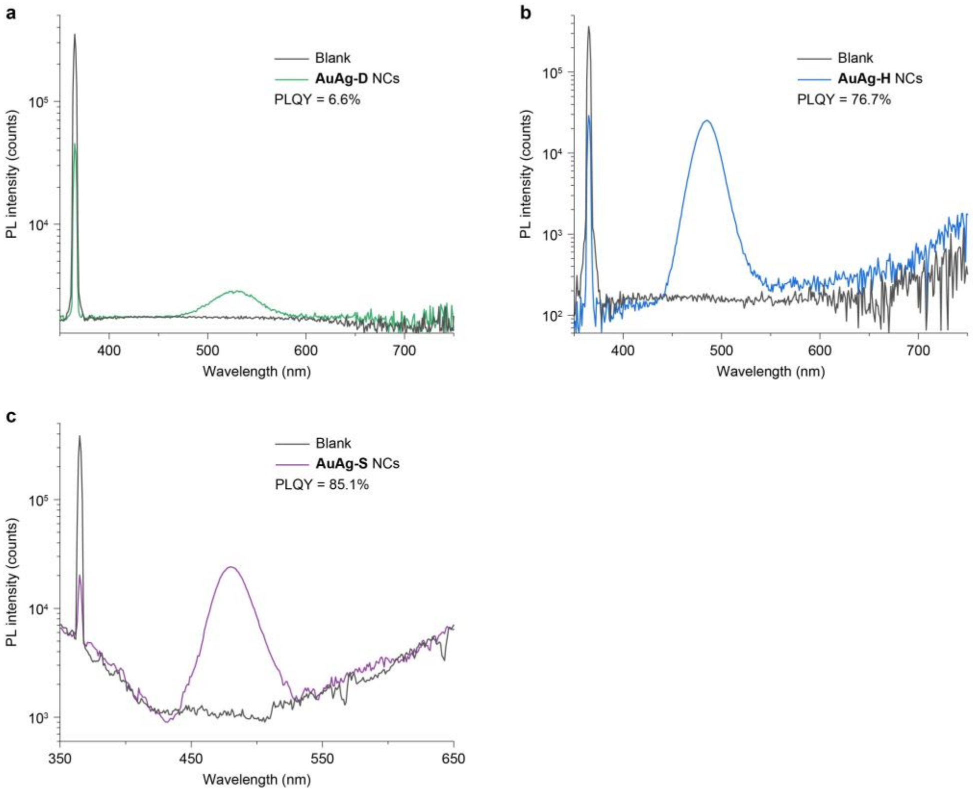

Supplementary Fig. 3. Absolute PLQY measurements. a-c PLQY measurements of AuAg-D, AuAg-H, and AuAg-S NCs by using an integrating sphere. The excitation wavelength is 365 nm for all NCs.

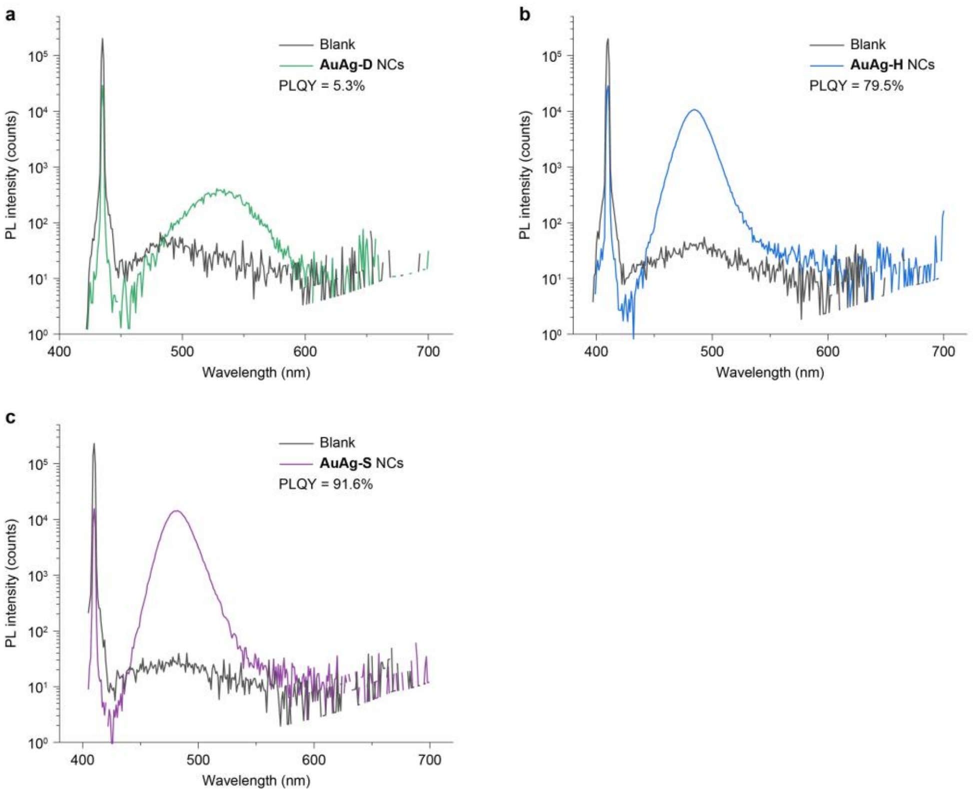

Supplementary Fig. 4. Near-bandgap excited PLQY measurements. a-c Near-bandgap excited PLQY measurements of AuAg-D, AuAg-H, and AuAg-S NCs by using an integrating sphere. The excitation wavelength is 435 nm for AuAg-D NCs, and 410 nm for AuAg-H and AuAg-S NCs, respectively.

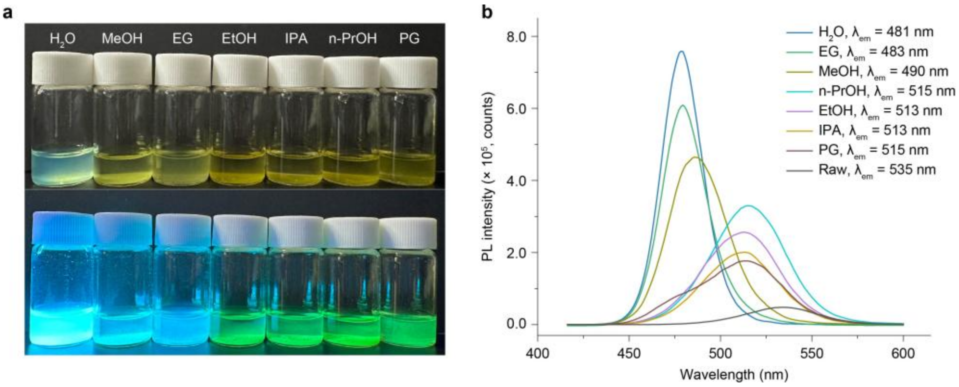

Supplementary Fig. 5. Universal validation with different alcohols. a Digital photos show AuAg-D NCs added with different alcohols under sunlight (top plane) and 365 nm near-UV light illumination (bottom plane). The permittivity (ε) of H_2O is 78.5. MeOH: methanol (ε = 32.6); EG: ethylene glycol (ε = 37.7); EtOH: ethanol (ε = 24.3); IPA: isopropanol (ε = 19.9); n-PrOH: n-propanol (ε = 20.1); PG: 1,2-propylene glycol (ε = 32.0). **b** PL spectra of raw AuAg-D NCs and AuAg-D NCs added with different alcohols. The excitation wavelength was set at 365 nm. The samples were prepared by injecting 5 mL alcohol solvent into translucent scintillation vials with ~3 mg AuAg-D NCs powder. All of the above operations are completed in a glove box in a N_2 atmosphere.

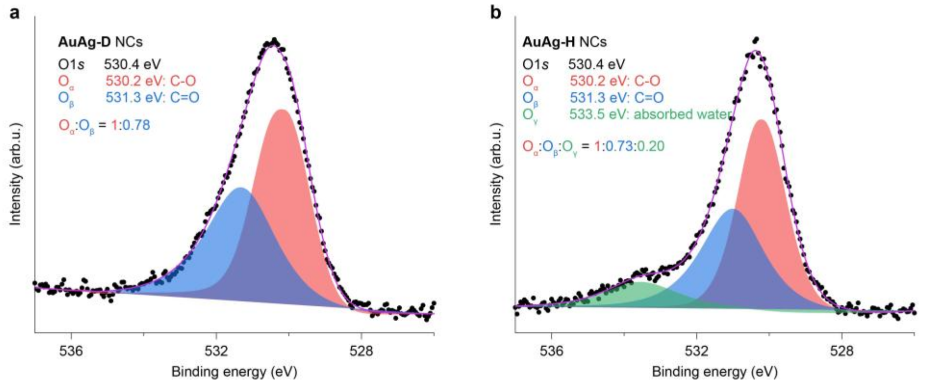

Supplementary Fig. 6. O 1*s* XPS analysis. a, **b** High-resolution O 1*s* XPS spectra of AuAg-D and AuAg-H NCs and their corresponding peak deconvolutions. The nearly consistent ratio between O_α and O_β in AuAg-D (1:0.78) and AuAg-H (1:0.73) NCs demonstrates the appearance of O_γ is not transformed from O_α and O_β components.

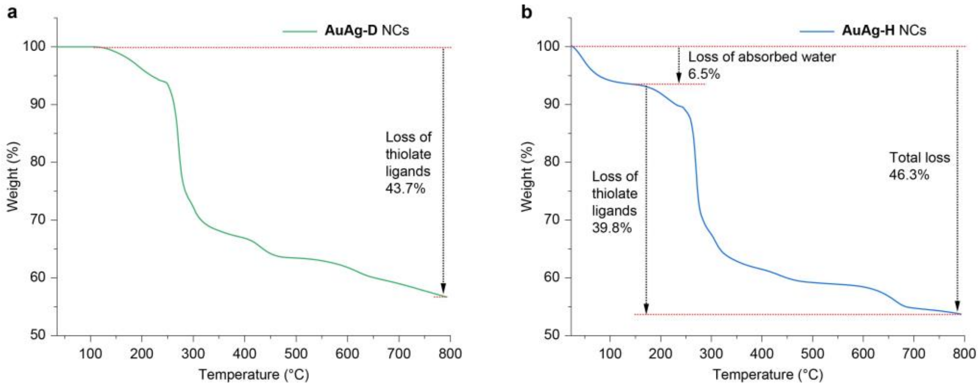

Supplementary Fig. 7. TGA measurements. a, **b** TGA results of AuAg-D and AuAg-H NCs.

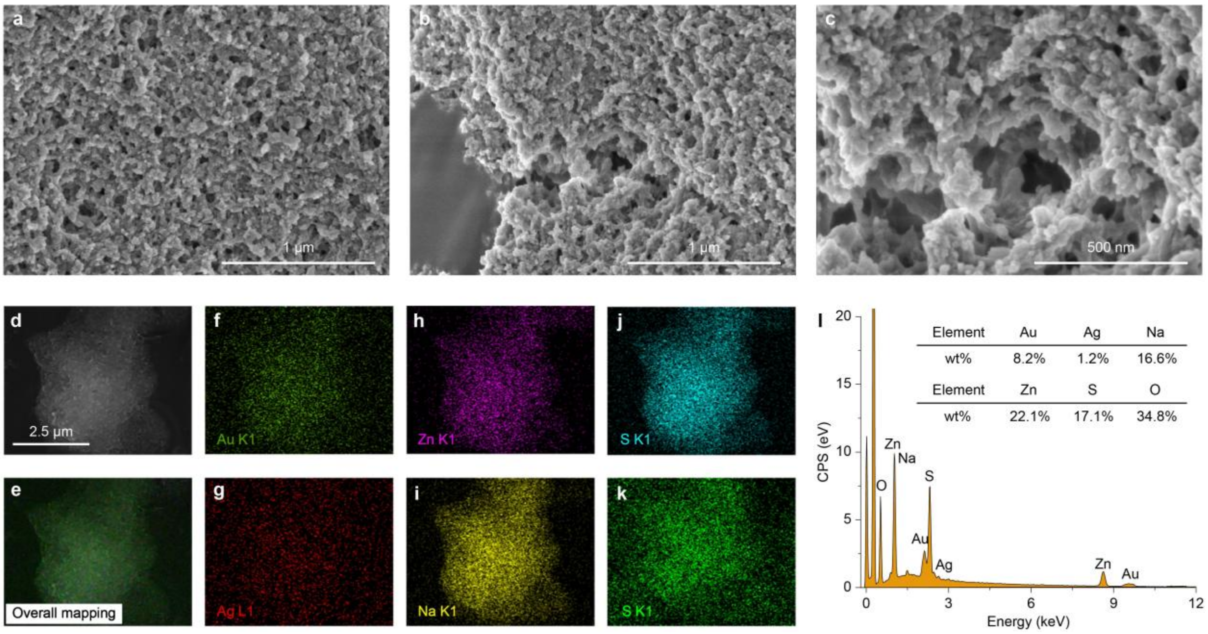

Supplementary Fig. 8. Micromorphology and elemental characterizations. a-c SEM images of AuAg-D NCs recorded under different magnifications. **d-k** Distribution of constituent elements in AuAg-D NCs. **l** EDS spectra and elemental mass ratio statistics of AuAg-D NCs. EDS mappings demonstrate that all constituent elements are uniformly distributed in AuAg NCs powder.

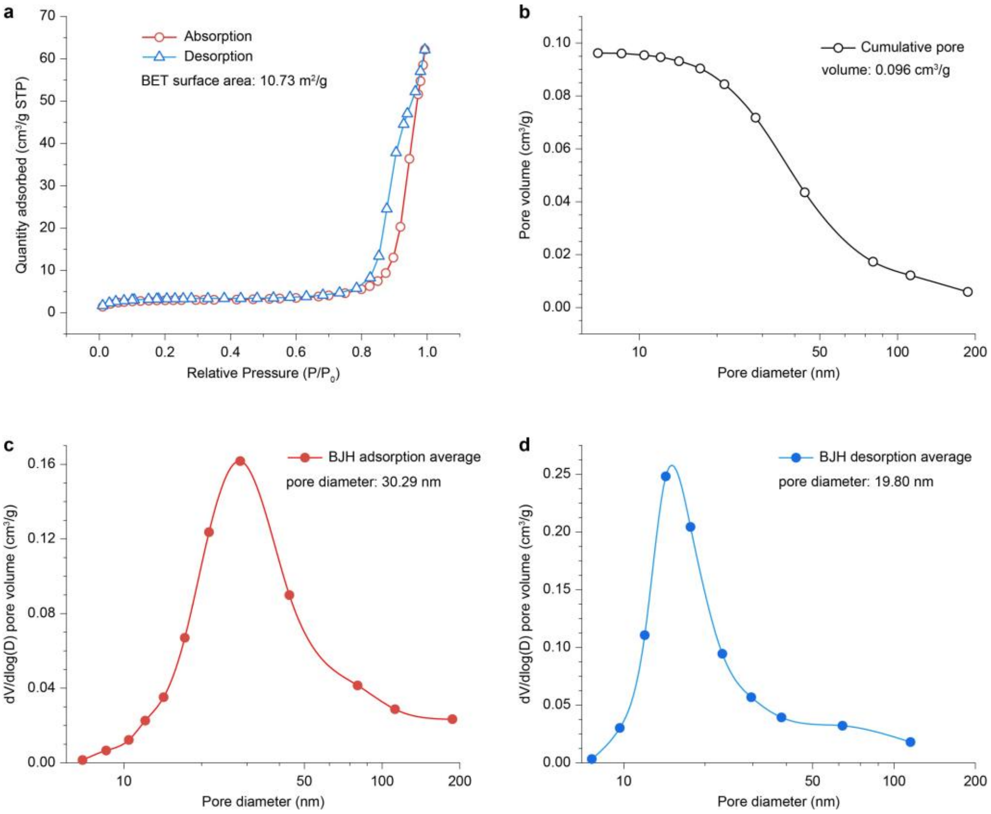

Supplementary Fig. 9. BET analysis. a N_2 adsorption/desorption isotherms at 77 K (P_0 is atmospheric pressure). **b** BJH adsorption cumulative pore volume. **c** BJH adsorption dV/dlog(D) pore volume. **d** BJH ddsorption dV/dlog(D) pore volume.

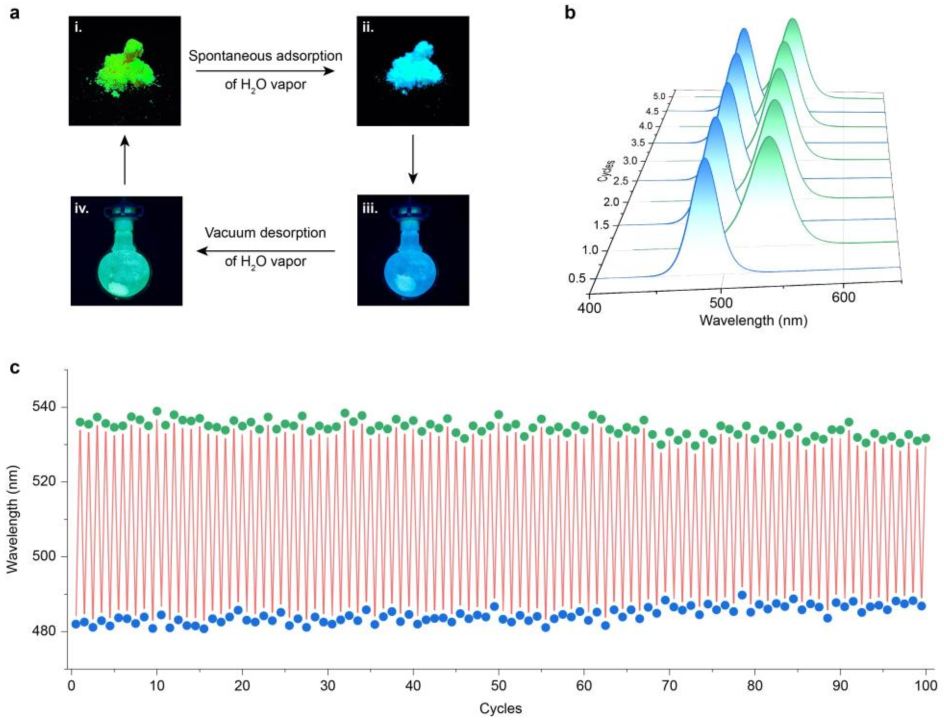

Supplementary Fig. 10. Reproducibility validation of water hydration-dehydration of AuAg NCs. a Digital photos show the change in emitting color of AuAg NCs under 365 nm near-UV light irradiation during one cycle of spontaneous water absorption in air and vacuum dehydration. **b** PL spectra of AuAg NCs under 365 nm excitation in five hydration-dehydration cycles. **c** Variation of the emission peak position of AuAg NCs during 100 hydration-dehydration cycles. The corresponding water-induced changes in emitting color in the hydration/dehydration processes are supplied in Supplementary Video 1 and 2, respectively.

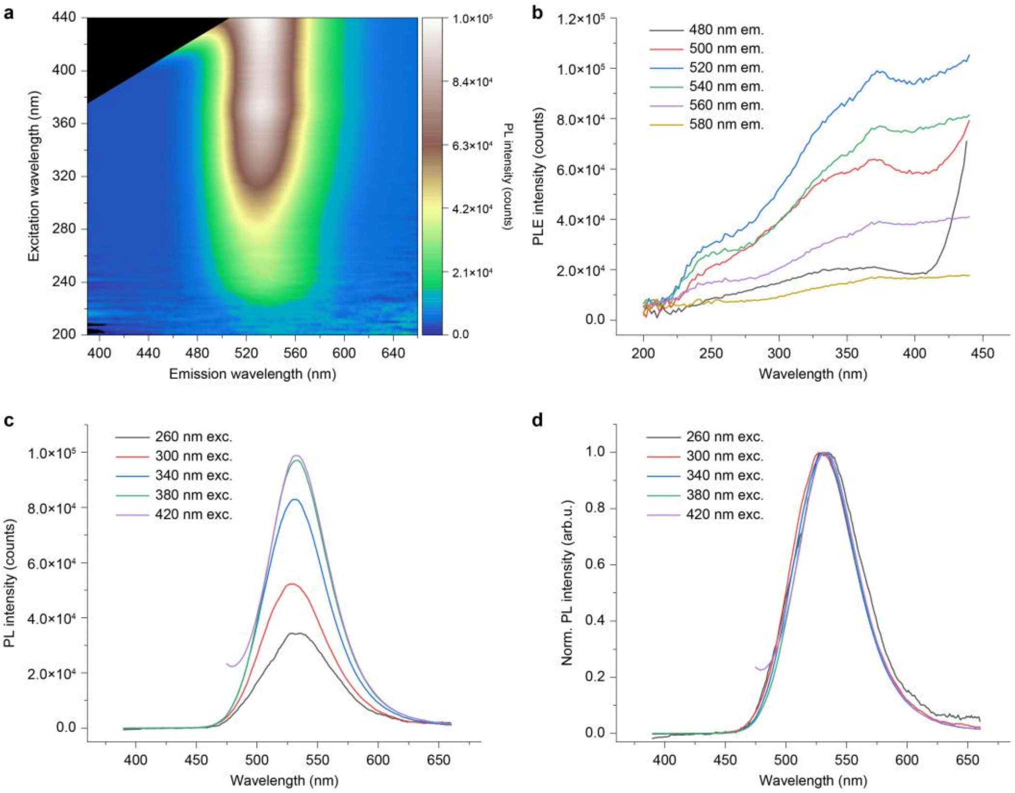

Supplementary Fig. 11. Excitation-dependent emission property of AuAg-D NCs. a Excitation-emission contour map of AuAg-D NCs. **b** PLE spectra upon monitoring the emission wavelength from 480 to 580 nm with a wavelength interval of 20 nm. **c** PL spectra upon 260-420 nm excitation with a wavelength interval of 40 nm. **d** The corresponding normalized PL spectra in **c**.

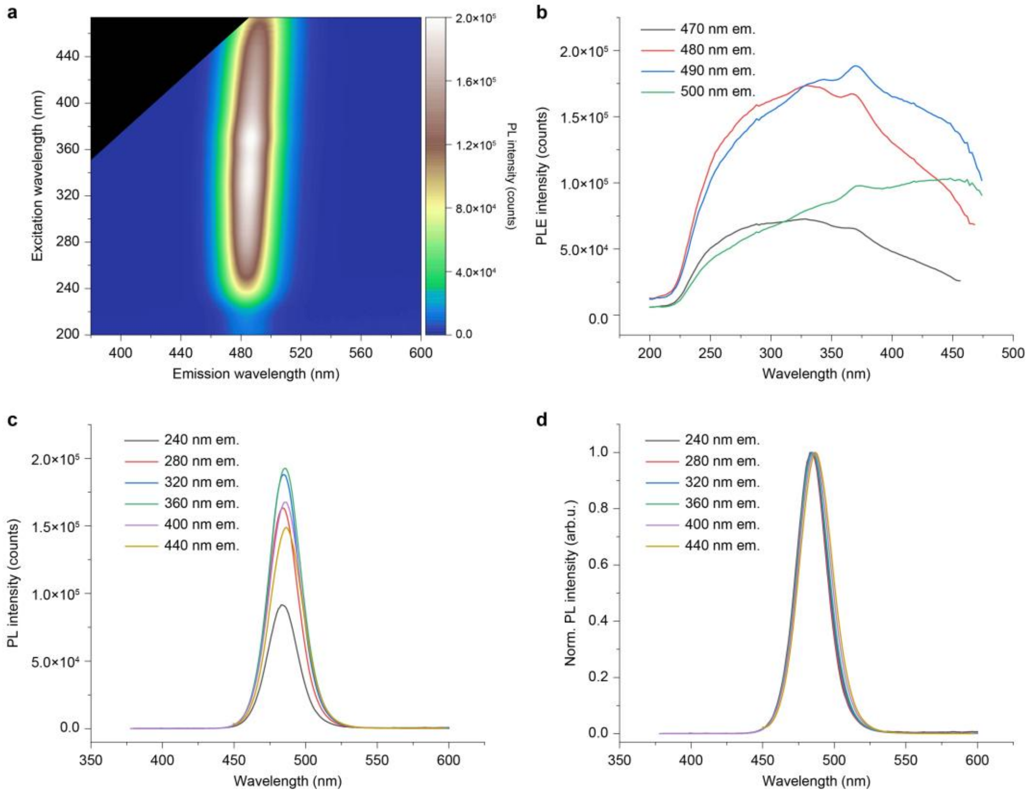

Supplementary Fig. 12. Excitation-dependent emission property of AuAg-H NCs. a Excitation-emission contour map of AuAg-H NCs. **b** PLE spectra upon monitoring the emission wavelength from 470 to 500 nm with a wavelength interval of 10 nm. **c** PL spectra upon 240-440 nm excitation with a wavelength interval of 40 nm. **d** The corresponding normalized PL spectra in **c**.

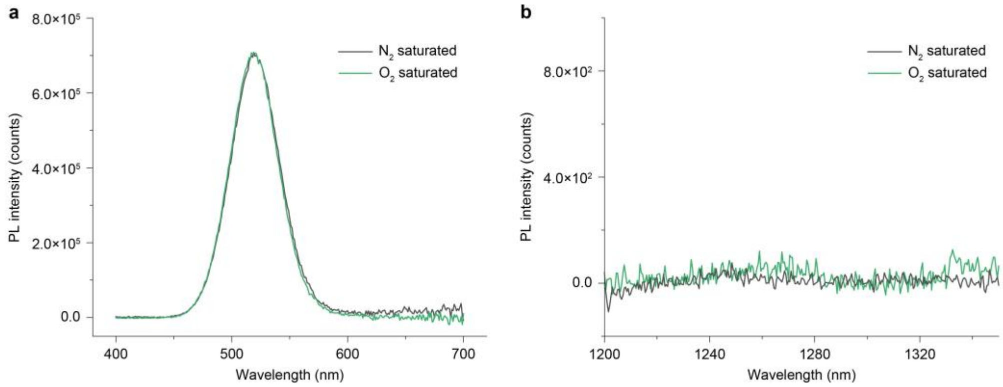

Supplementary Fig. 13. Validation of phosphorescence in AuAg-D NCs. a PL spectra of N_2- and O_2-saturated AuAg-D NCs powders upon 365 nm excitation. **b** NIR PL spectra of N_2- and O_2-saturated AuAg-D NCs powders.

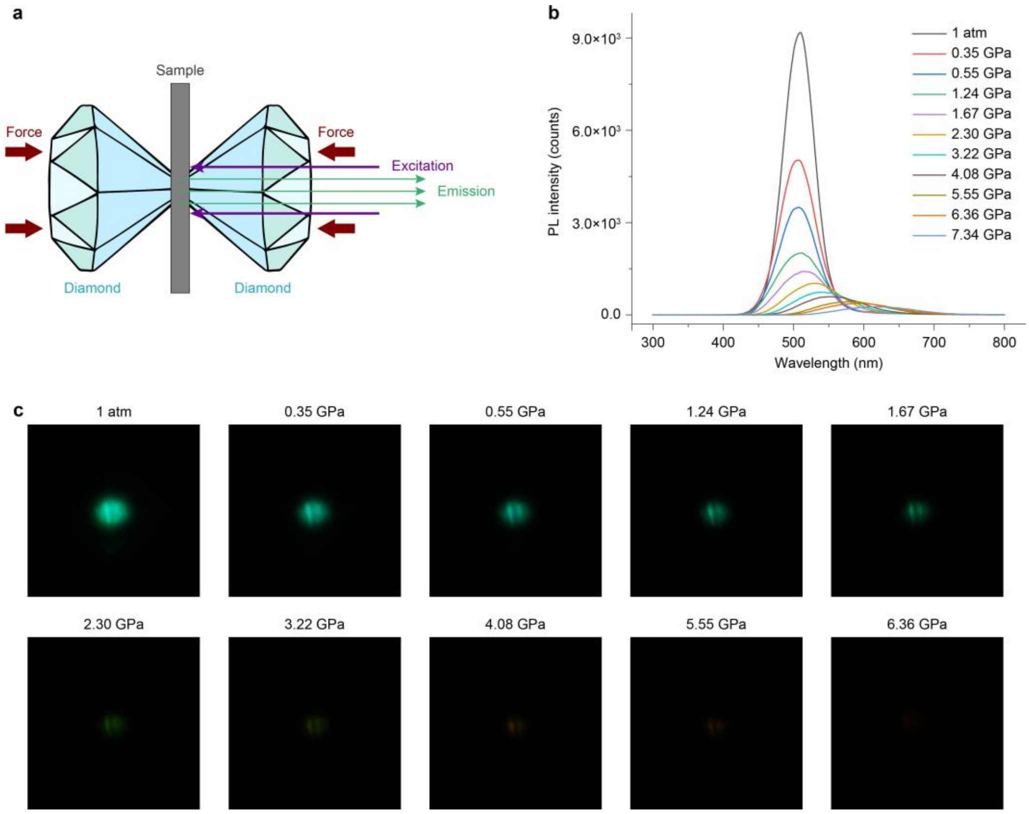

Supplementary Fig. 14. *In situ* high-pressure PL analysis. a Schematic illustration of the apparatus for the *in situ* measurement of high-pressure PL using a DAC device. **b** PL spectra of AuAg-D NCs under different pressures. The excitation wavelength is fixed at 355 nm. **c** The corresponding digital photographs show the variation of the emitting color and intensity of AuAg-D NCs under different pressures.

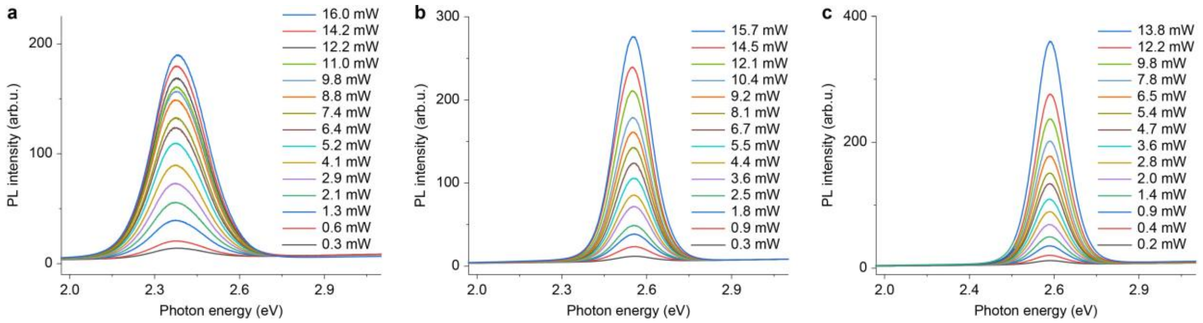

Supplementary Fig. 15. Validation of trap-state emission. a-c PL spectra of AuAg-D, AuAg-H, and AuAg-S NCs recorded under different excitation powers.

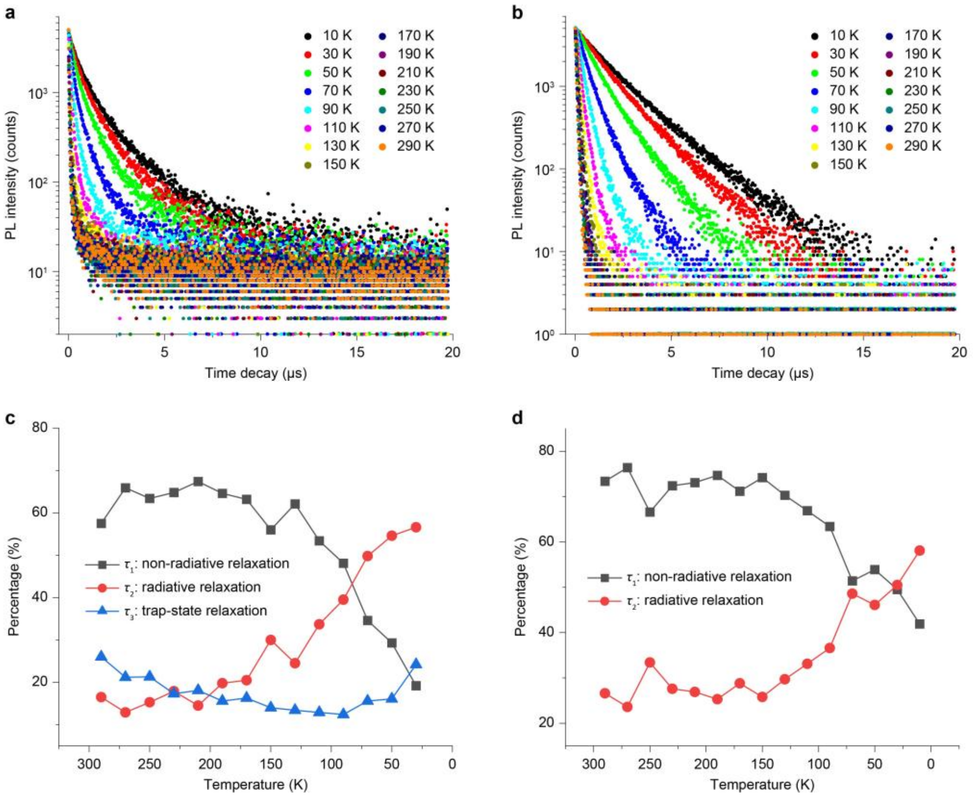

Supplementary Fig. 16. Temperature-dependent PL lifetime. a, **b** PL lifetimes of AuAg-D and AuAg-H NCs collected from 10 to 290 K with a temperature interval of 20 K. A 370 nm pulsed laser was used as the excitation source and the monitoring wavelength was set at 536 and 482 nm for AuAg-D and AuAg-H NCs samples, respectively. **c**, **d** Variations of τ_1, τ_2, and τ_3 in the PL decays of AuAg-D NCs and τ_1, τ_2 in the PL decays of AuAg-H NCs with the decrease of temperature.

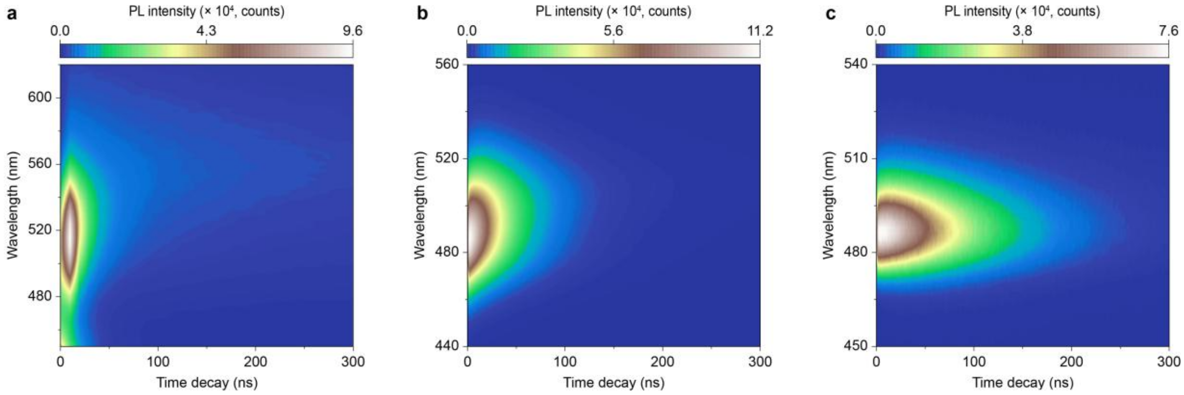

Supplementary Fig. 17. TRPL analysis. a-c TRPL maps of AuAg-D, AuAg-H, and AuAg-S NCs. A 370 nm pulsed laser was used as the excitation source.

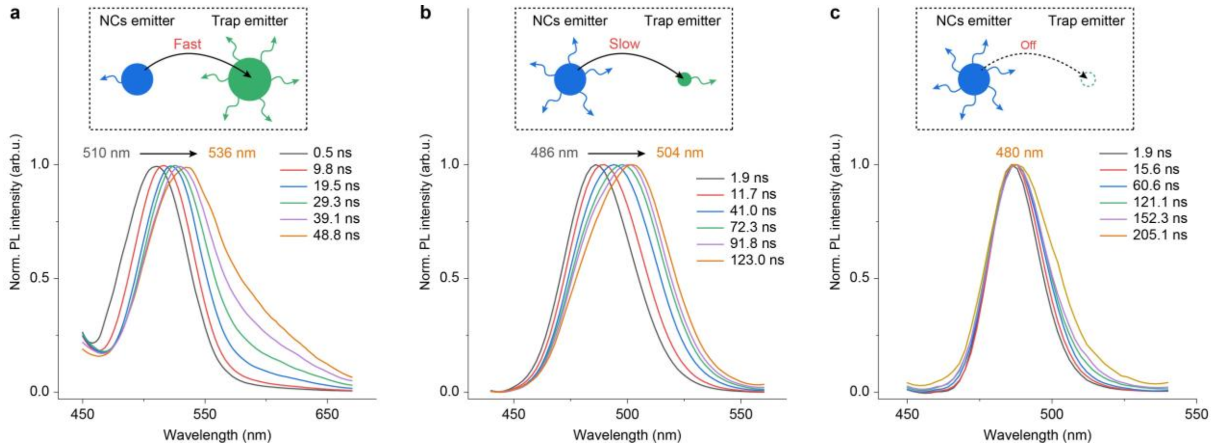

Supplementary Fig. 18. TRPL analysis. **a-c** Normalized TRPL spectra of AuAg-D, AuAg-H, and AuAg-S NCs in 48.8, 123.0, and 205.1 ns, respectively. The peaks of the PL spectra at the beginning and end of the delay time are marked for clearer identification of the shift of the PL peaks. The insets in **a-c** illustrate the different electron transfer rates in three AuAg NCs informed from the shift range of the PL peak.

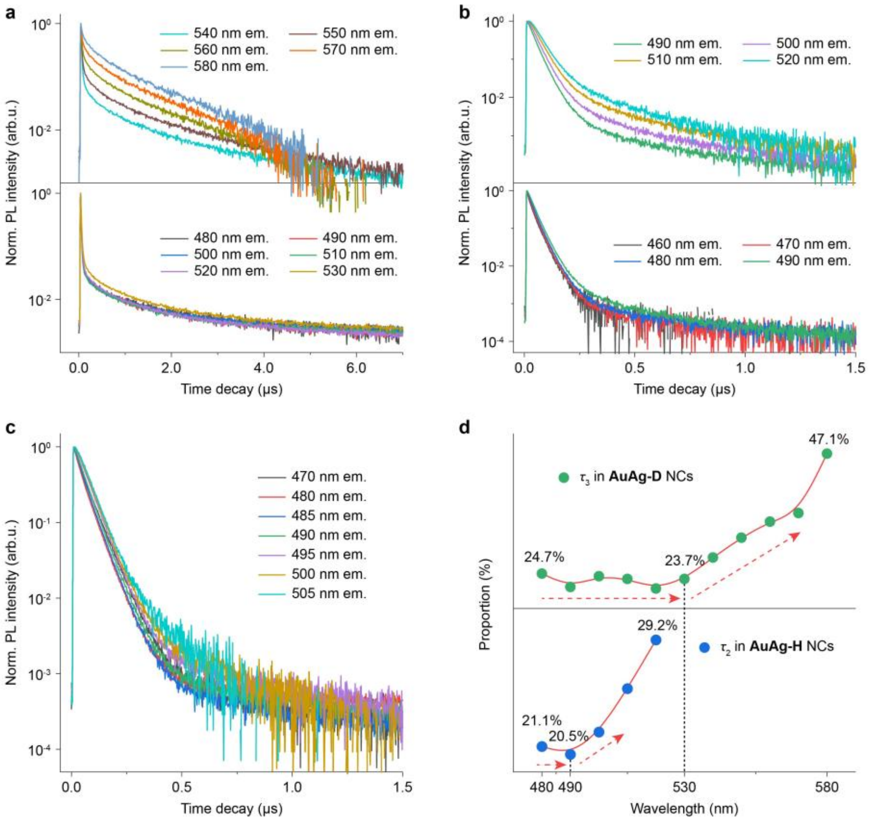

Supplementary Fig. 19. PL lifetimes at different monitoring wavelengths. a Normalized PL lifetimes of AuAg-D NCs in the monitoring wavelength of 480-580 nm. **b** Normalized PL lifetimes of AuAg-H NCs in the monitoring wavelength of 460-520 nm. **c** Normalized PL lifetimes of AuAg-S NCs in the monitoring wavelength of 470-505 nm. **d** The variations of the long-time component in the PL decays (τ_3 in AuAg-D NCs and τ_2 in AuAg-H NCs) with the change of monitoring wavelength.

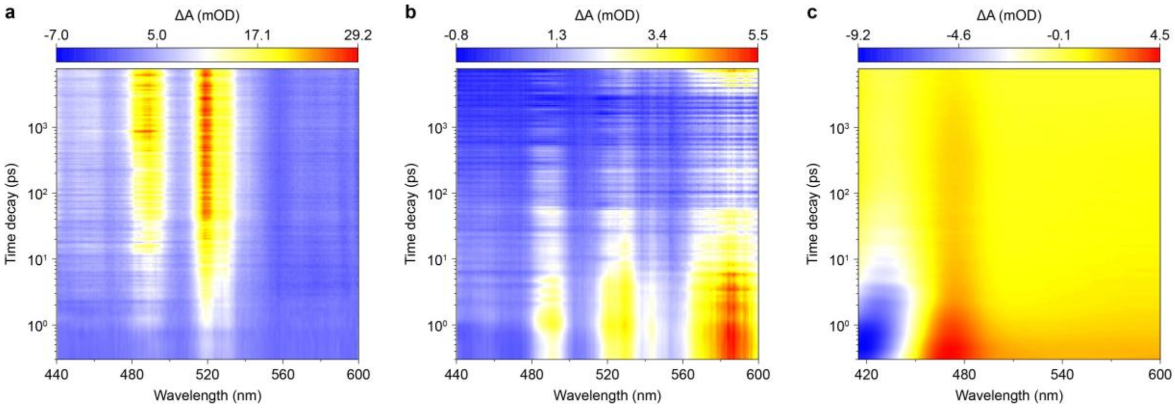

Supplementary Fig. 20. Femtosecond-TA results. a-c Femtosecond-TA maps of AuAg-D, AuAg-H, and AuAg-S NCs upon 365 nm laser pump. Symbol: ΔA is the change in absorption intensity in units of optical density.

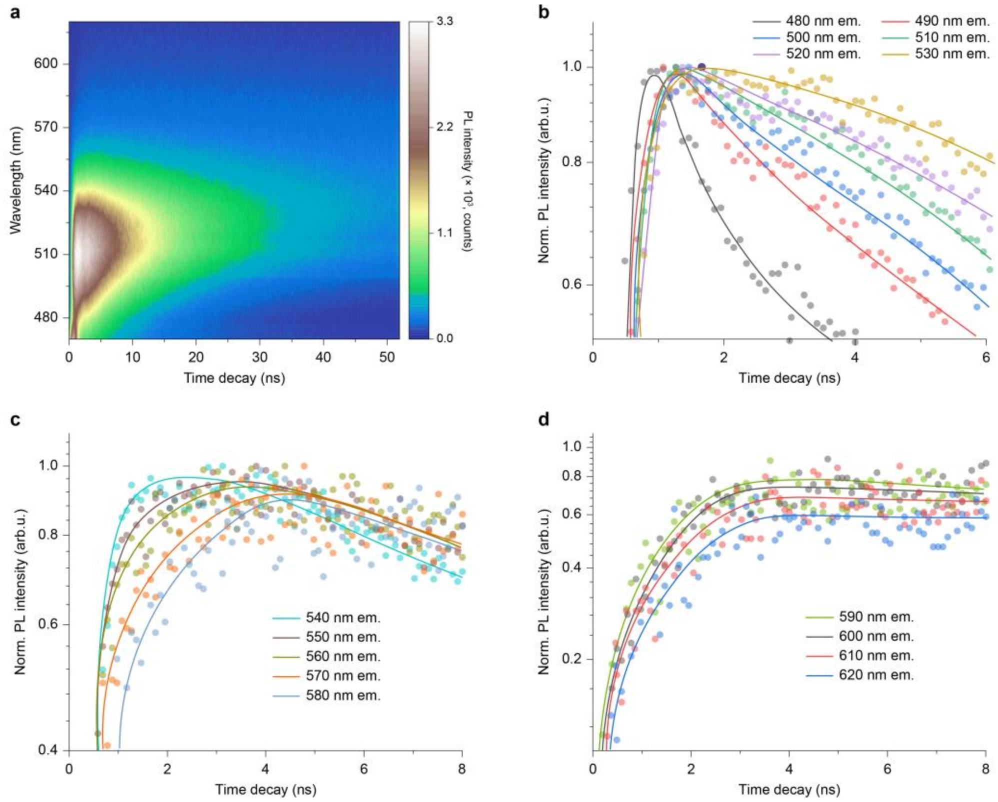

Supplementary Fig. 21. Short-time TRPL result of AuAg-D NCs. a Short-time TRPL map of AuAg-D NCs upon 370 nm laser excitation. **b-d** Normalized PL decays in the monitoring wavelength range of 480-530 nm, 540-580 nm, and 590-620 nm, respectively.

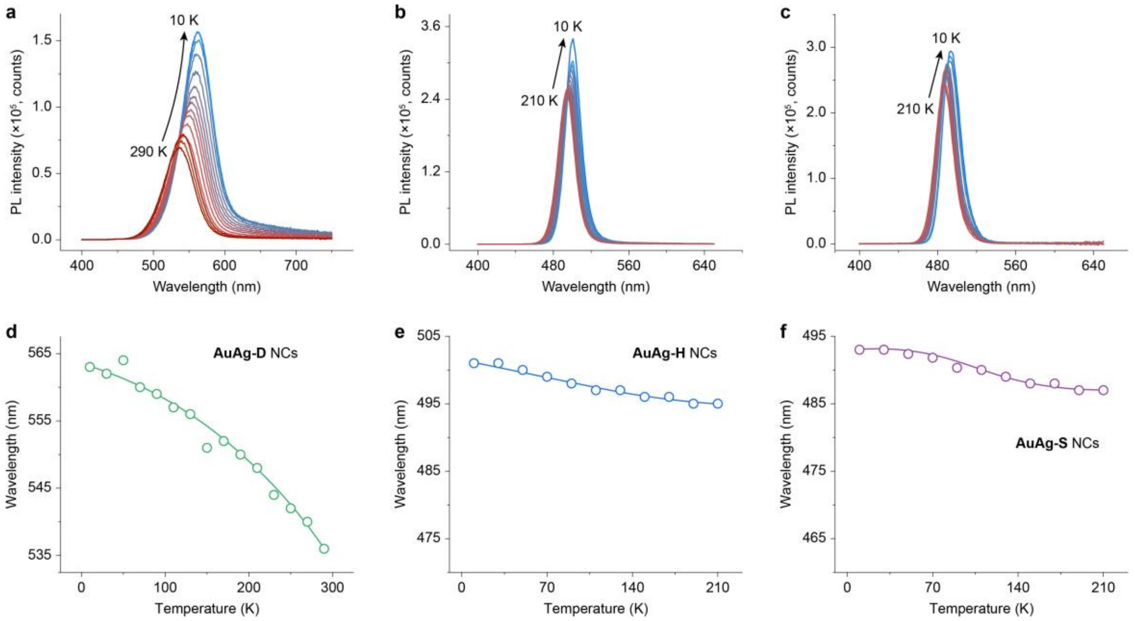

Supplementary Fig. 22. Low-temperature PL analysis. a-**c** Temperature-dependent PL spectra of AuAg-D, AuAg-H, and AuAg-S NCs. **d**-**f** Plots of the PL peak of AuAg-D, AuAg-H, and AuAg-S NCs as a function of temperature.

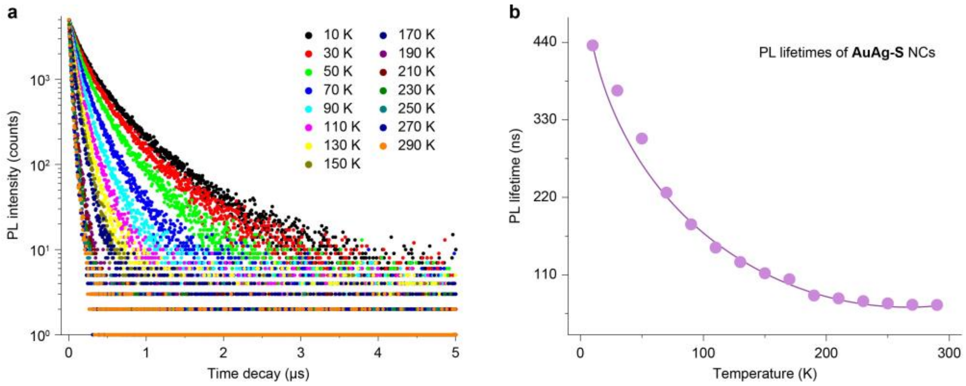

Supplementary Fig. 23. Temperature-dependent PL lifetime of AuAg-S NCs. a PL lifetimes of AuAg-S NCs collected from 10 to 290 K with a temperature interval of 20 K. A 370 nm pulsed laser was used as the excitation source and the monitoring wavelength was set at 480 nm. **b** Variation of the average PL lifetime of AuAg-S NCs under different testing temperatures.

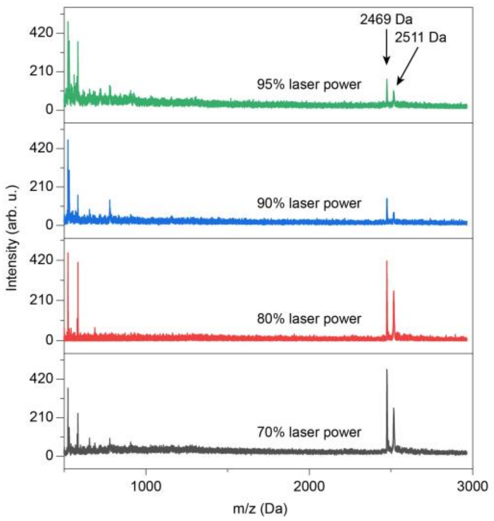

Supplementary Fig. 24. Positive MALDI-TOF mass spectra of AuAg-S NCs recorded under different laser powers. DCTB was used as the matrix for the test of all samples. With the increase of laser power from 70% to 95%, the mass signal at 2469 and 2511 Da gradually weakened while the mass signals between 400-700 Da were enhanced. These results confirm the assignments of the mass signal around 2500 Da and 400-700 Da to the intact AuAg NCs of $Au_8Ag_2(MPA)_6$-2H+2Na (2469 Da) and $Au_8Ag_2(MPA)_6$-2H+Zn+Na (2511 Da) and their fragmentations, respectively, because that the laser with higher power could break up the structure of the intact AuAg NCs and therefore generate more fragmentations.

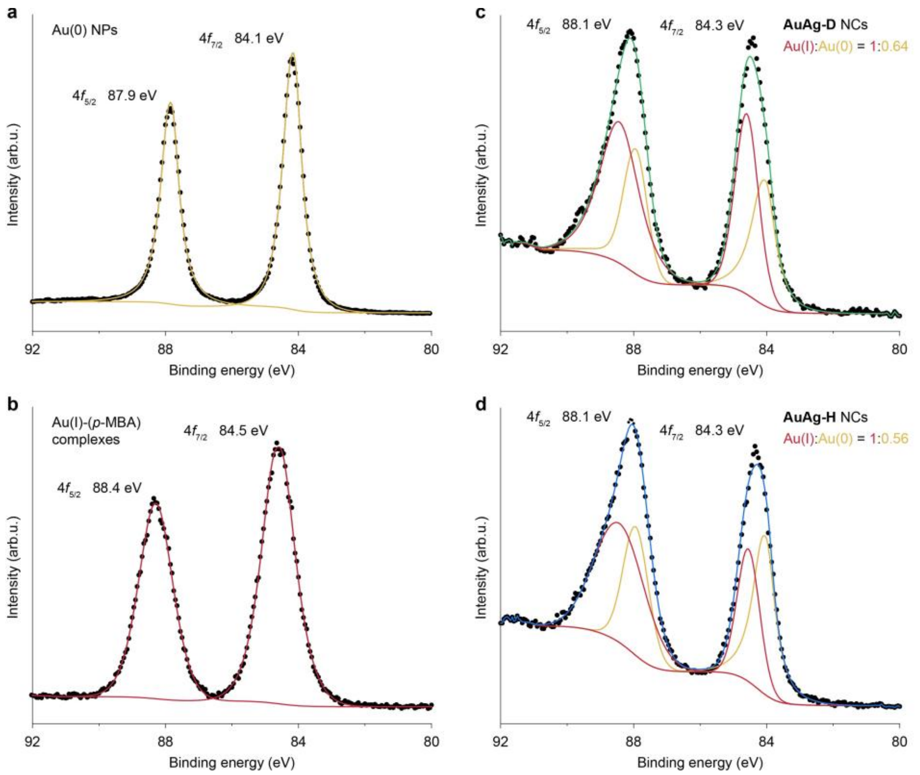

Supplementary Fig. 25. Au 4*f* XPS analysis. a, **b** High-resolution Au 4*f* XPS spectra of Au(0) NPs and Au(I)-(*p*-MBA) complexes. **c**, **d** High-resolution Au 4*f* XPS spectra of AuAg-D and AuAg-H NCs, and their corresponding peak deconvolutions using the Au 4*f* XPS spectra of Au(0) NPs and Au(I)-(*p*-MBA) complexes as the standard Au(0) and Au(I) XPS signals.

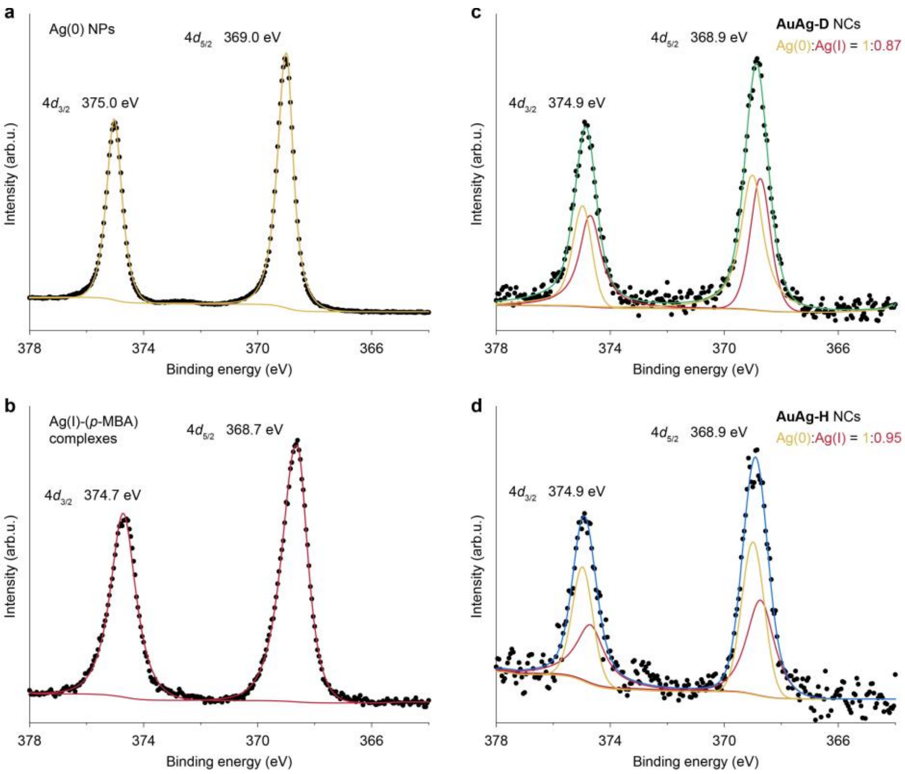

Supplementary Fig. 26. Ag 4*d* XPS analysis. a, **b** High-resolution Ag 4*d* XPS spectra of Ag(0) NPs and Ag(I)-(*p*-MBA) complexes. **c**, **d** High-resolution Ag 4*d* XPS spectra of AuAg-D and AuAg-H NCs, and their corresponding peak deconvolutions using the Ag 4*d* XPS spectra of Ag(0) NPs and Ag(I)-(*p*-MBA) complexes as the standard Ag(0) and Ag(I) XPS signals.

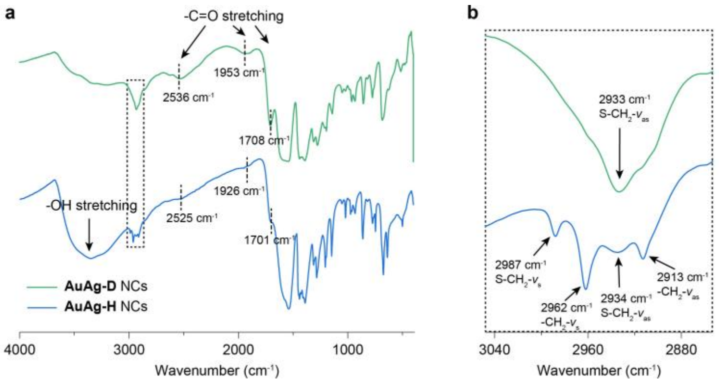

Supplementary Fig. 27. FTIR analysis. a FTIR spectra of AuAg-D and AuAg-H NCs. **b** The amplified FTIR spectra of AuAg-D and AuAg-H NCs in the wavenumber range of 3047-2853 cm^{-1}. The red shift of stretching vibration of -C=O group from AuAg-D to AuAg-H NCs implies the formation of hydrogen bondings between H_2O molecules and the -C=O group in MPA ligands. In addition, the splitting of methylene stretching vibrational peak at 2933 cm^{-1} in AuAg-D NCs into 2987, 2962, 2934, and 2913 cm^{-1} subpeaks in AuAg-H NCs is attributed to the vibration coupling of adjacent methylene groups in MPA ligands induced by the newly formed hydrogen bondings[1].

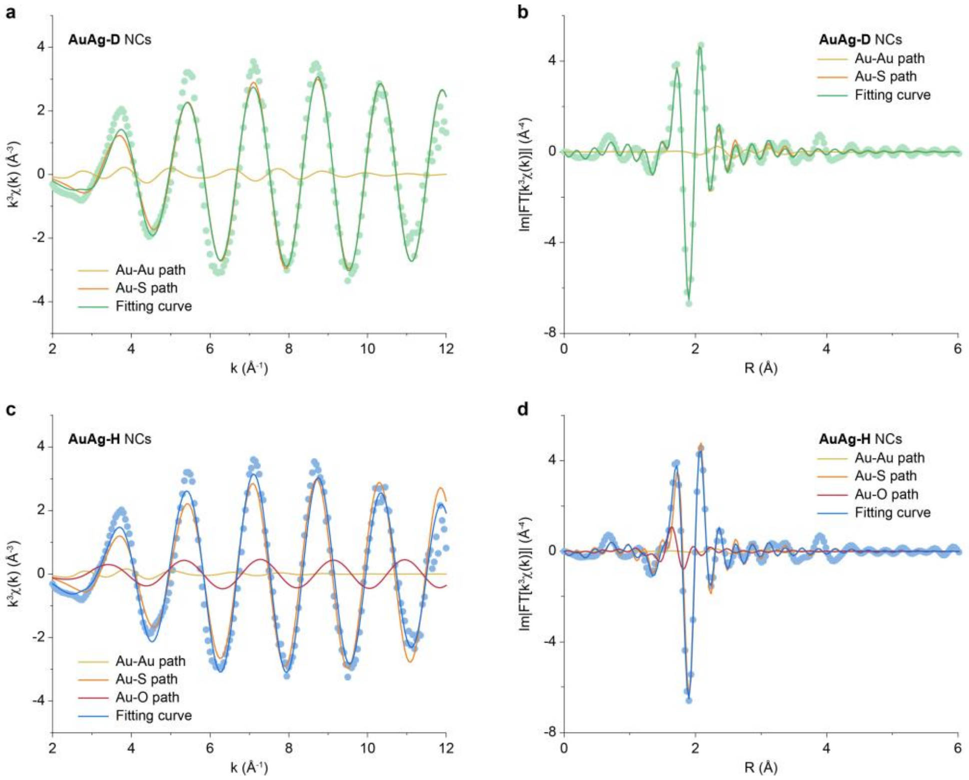

Supplementary Fig. 28. Au L_3-edge EXAFS data analysis of AuAg-D and AuAg-H NCs. **a**, **c** The experimental data and corresponding best fits of Au L_3-edge EXAFS of AuAg-D and AuAg-H NCs, respectively, shown in k^3-weighted *k*-space. **b**, **d** The experimental data and corresponding best fits of Au L_3-edge Fourier transformed (FT)-EXAFS (FT-EXAFS) of AuAg-D and AuAg-H NCs, respectively, shown in k^3-weighted *R*-space (imaginary component).

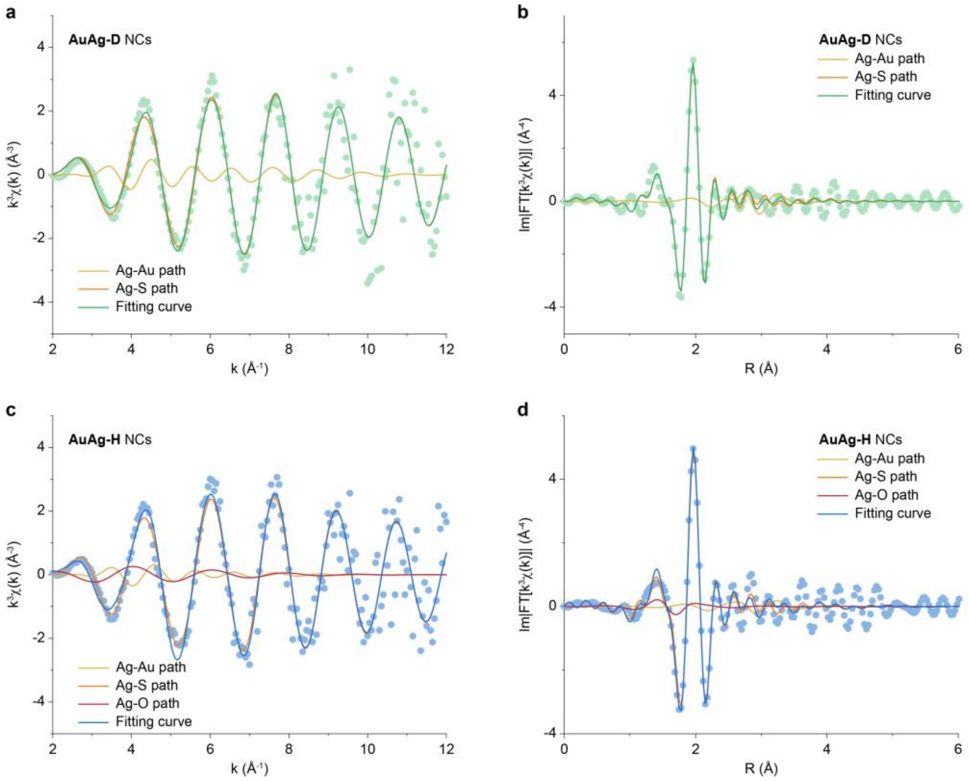

Supplementary Fig. 29. Ag K-edge EXAFS data analysis of AuAg-D and AuAg-H NCs. a, **c** The experimental data and corresponding best fits of Ag K-edge EXAFS of AuAg-D and AuAg-H NCs, respectively, shown in k^3-weighted *k*-space. **b**, **d** The experimental data and corresponding best fits of Ag K-edge FT-EXAFS of AuAg-D and AuAg-H NCs, respectively, shown in k^3-weighted *R*-space (imaginary component).

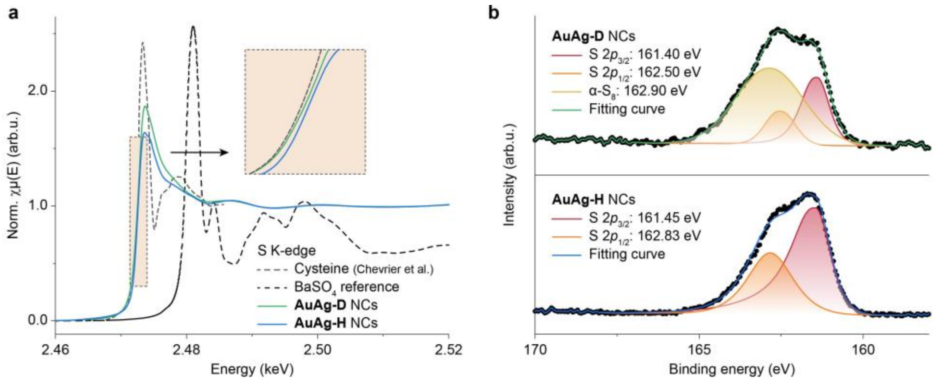

Supplementary Fig. 30. Local structure of S atoms in AuAg-D and AuAg-H NCs. a S K-edge XANES spectra of Cysteine, $BaSO_4$, AuAg-D, and AuAg-H NCs. Inset shows the magnified XANES spectra in the energy range of 2.471-2.474 keV. The S K-edge XANES spectra of Cysteine are extracted from Chevrier et al. (2020)[2]. Reprinted with permission from American Chemical Society. **b** High-resolution S 2*p* XPS spectra of AuAg-D and AuAg-H NCs, and their corresponding peak deconvolutions. The ratio of integrated peak area for S $2p_{3/2}$ and S $2p_{1/2}$ was fixed at 2:1 in both peak deconvolutions. Note that both the most intense peaks in S K-edge XANES spectra of AuAg-D and AuAg-H NCs get broadened compared to that of free thiol ligands (Cysteine) owing to the formation of S-Au(Ag) interactions in staple motifs in AuAg NCs. The α-S_8 peak in the high-resolution S 2*p* XPS spectra of AuAg-D NCs is assigned to crystalline sulfur which usually occurs at 162.90 eV[3].

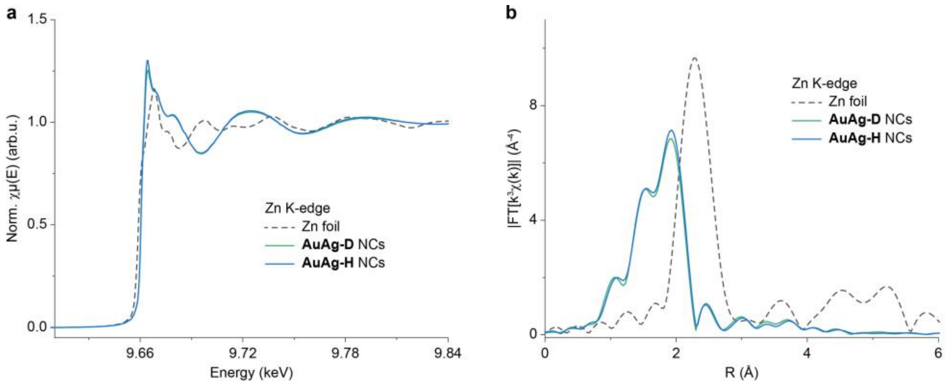

Supplementary Fig. 31. Local structure of Zn atoms in AuAg-D and AuAg-H NCs. a Zn K-edge XANES spectra of Zn foil, AuAg-D, and AuAg-H NCs. **b** Zn K-edge FT-EXAFS spectra of Zn foil, AuAg-D, and AuAg-H NCs, shown in k^3-weighted *R*-space.

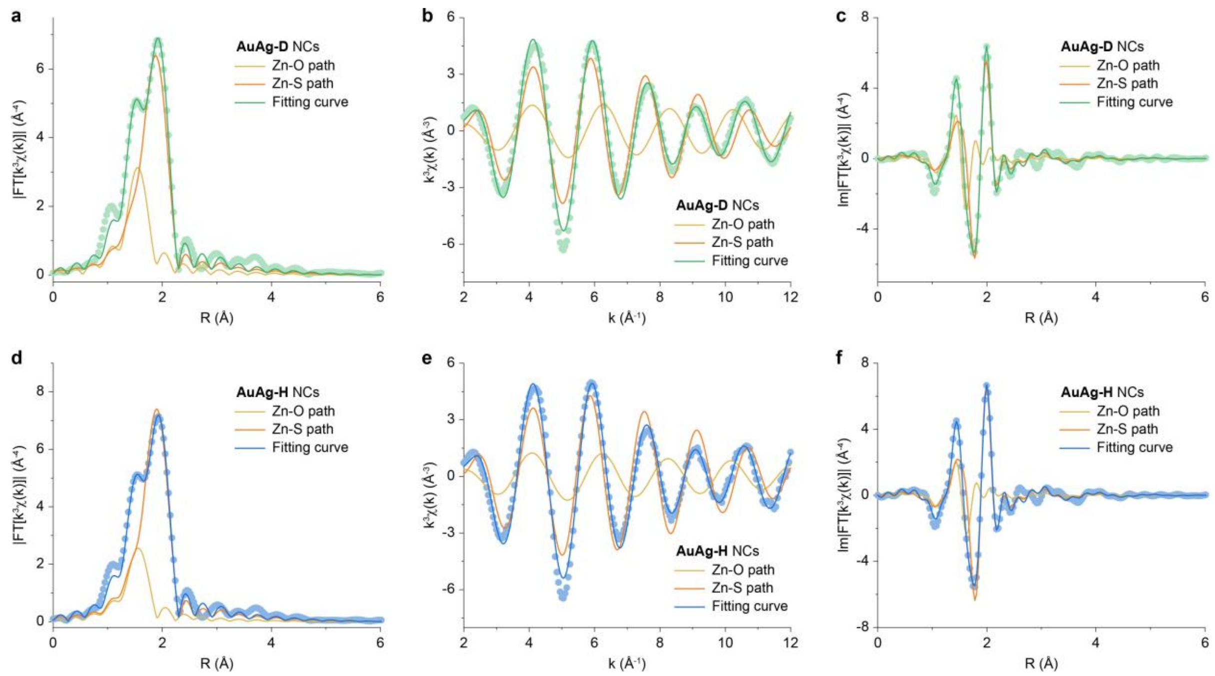

Supplementary Fig. 32. Zn K-edge EXAFS data analysis of AuAg-D and AuAg-H NCs. a, **d** The experimental data and corresponding best fits of Zn K-edge FT-EXAFS of AuAg-D and AuAg-H NCs, respectively, shown in k^3-weighted *R*-space. **b**, **e** The experimental data and corresponding best fits of Zn K-edge EXAFS of AuAg-D and AuAg-H NCs, respectively, shown in k^3-weighted *k*-space. **c**, **f** The experimental data and corresponding best fits of Zn K-edge FT-EXAFS of AuAg-D and AuAg-H NCs, respectively, shown in k^3-weighted *R*-space (imaginary component).

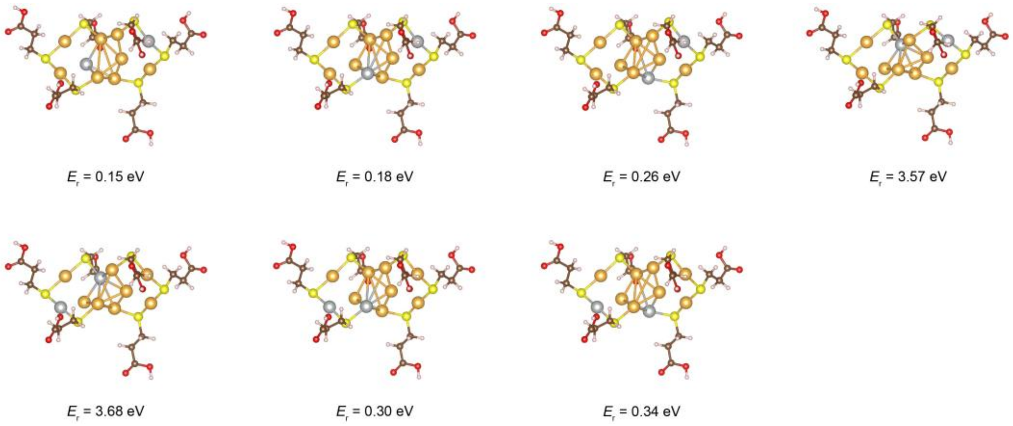

Supplementary Fig. 33. Structural optimization of Ag atom positions in AuAg-D NCs. The relative energies (E_r) of each model are given to gauge the thermodynamic stability of the model.

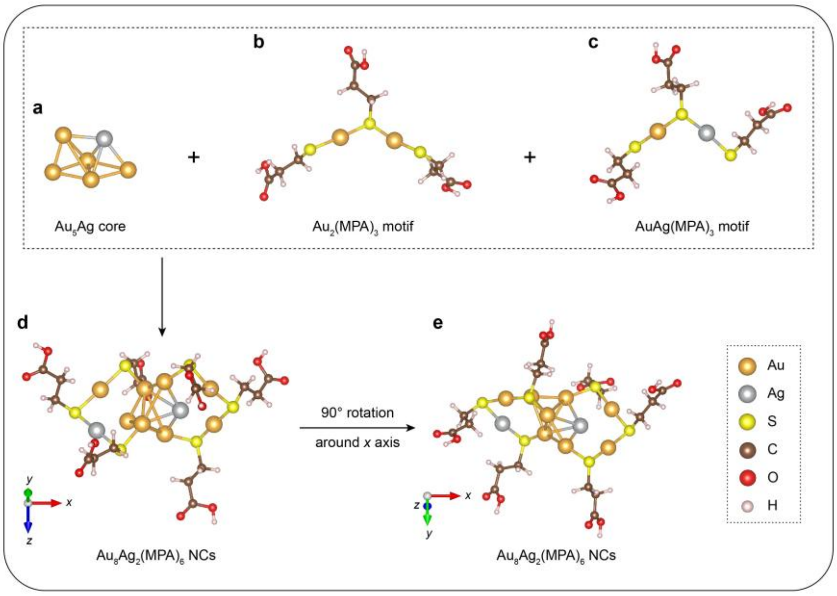

Supplementary Fig. 34. Structural anatomy of AuAg-D NCs. a The Au_5Ag core. **b** The $Au_2(MPA)_3$ staple motif. **c** The $AuAg(MPA)_3$ staple motif. **d** The whole Au_8Ag_2 cluster protected by 6 MPA ligands. **e** View of the $Au_8Ag_2(MPA)_6$ NCs by rotating around *x* axis for 90°.

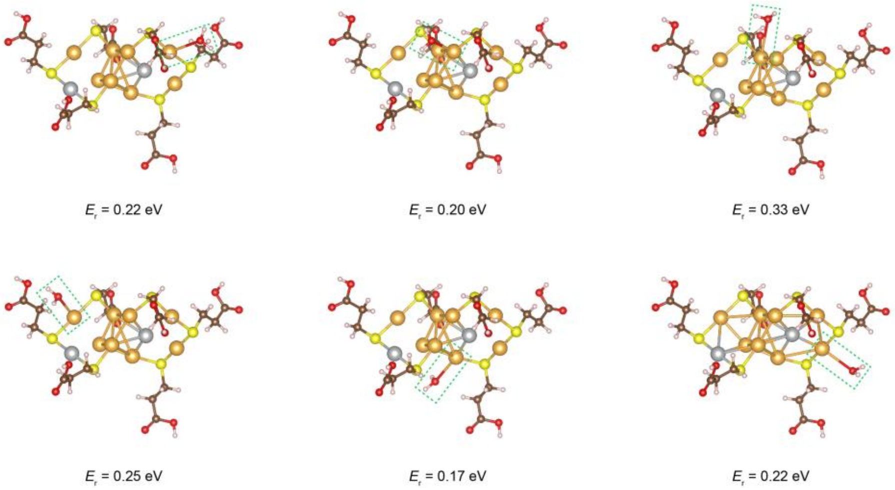

Supplementary Fig. 35. Structural optimization of the anchoring position of the absorbed H_2O molecule in AuAg-H NCs. The relative energies of each model are given to gauge their corresponding thermodynamic stability.

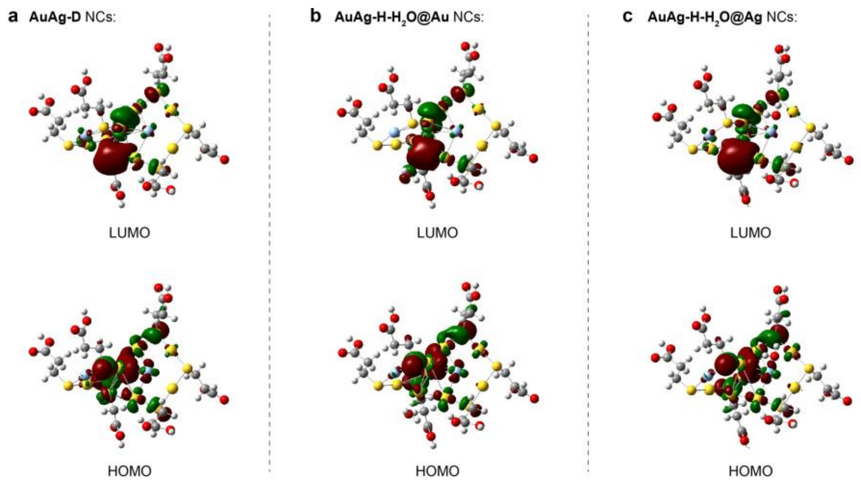

Supplementary Fig. 36. Comparison of the electron density profile of the HOMO and LUMO of three AuAg NCs. a AuAg-D NCs. **b** AuAg-H-H_2O@Au NCs. **c** AuAg-H-H_2O@Ag NCs.

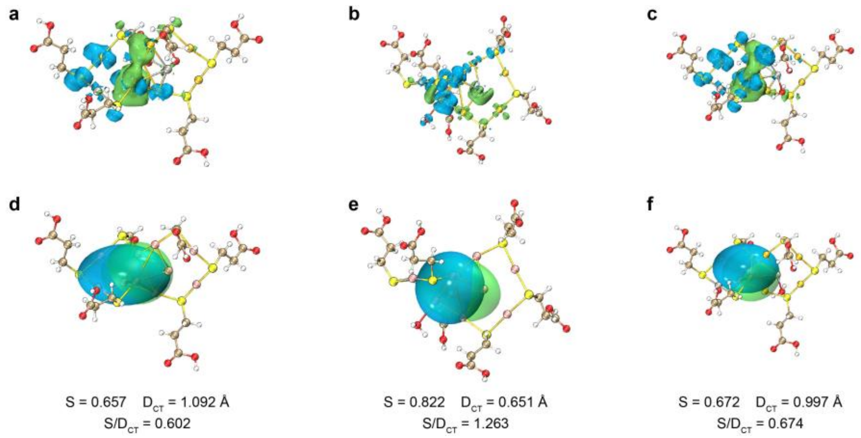

Supplementary Fig. 37. Quantitative hole-electron analysis of three AuAg NCs. a-c Computed charge density (CCD) difference between the ground and the excited states of the donor-accepter (D-A) pairs at an isovalue of 0.002 a.u. in AuAg-D, AuAg-H-H_2O@Au, and AuAg-H-H_2O@Ag NCs, respectively. The green and cyan represent increase and decrease in electron density, respectively. **d-f** Quantitative charge-transfer analysis based on the atomic dipole corrected Hirshfeld (ADCH) atomic charges in AuAg-D, AuAg-H-H_2O@Au, and AuAg-H-H_2O@Ag NCs, respectively.

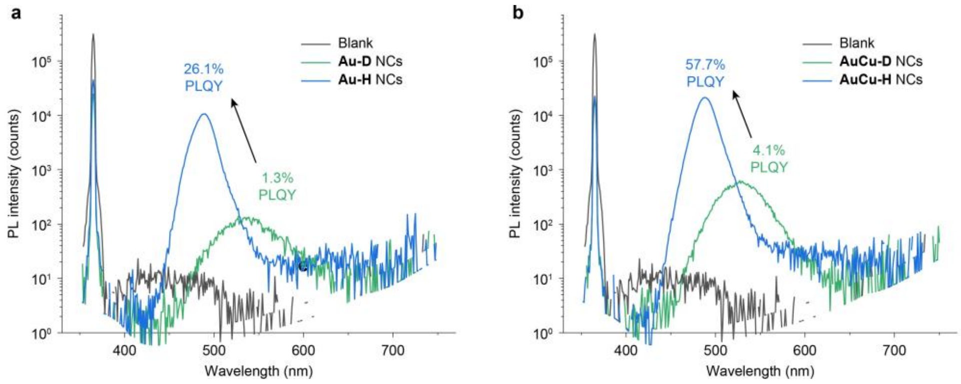

Supplementary Fig. 38. Absolute PLQY measurements. a PLQY measurements of Au-D and Au-H NCs. **b** PLQY measurements of AuCu-D and AuCu-H NCs. The excitation wavelength is set at 365 nm for all NCs and an integrating sphere on FLS1000 spectrofluorometer was using in the measurements.

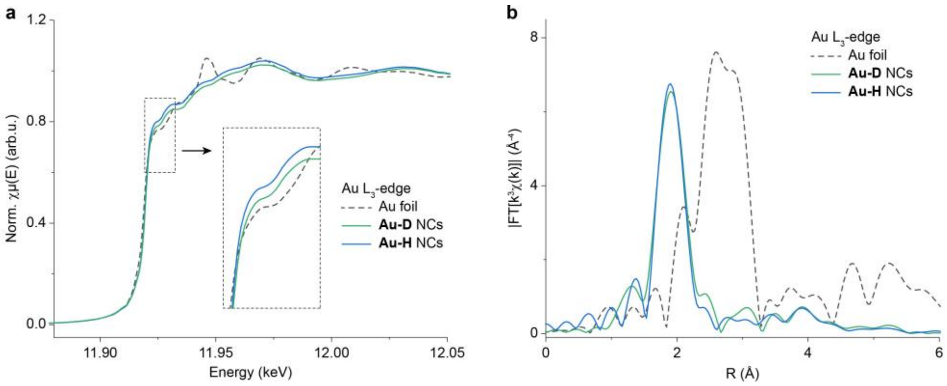

Supplementary Fig. 39. Local structure of Au atoms in Au-D and Au-H NCs. a Au L_3-edge XANES spectra of Au foil, Au-D, and Au-H NCs. Inset shows the magnified XANES spectra in the energy range of 11.919-11.933 keV. **b** Au L_3-edge FT-EXAFS spectra of Au foil, Au-D, and Au-H NCs, shown in k^3-weighted *R*-space.

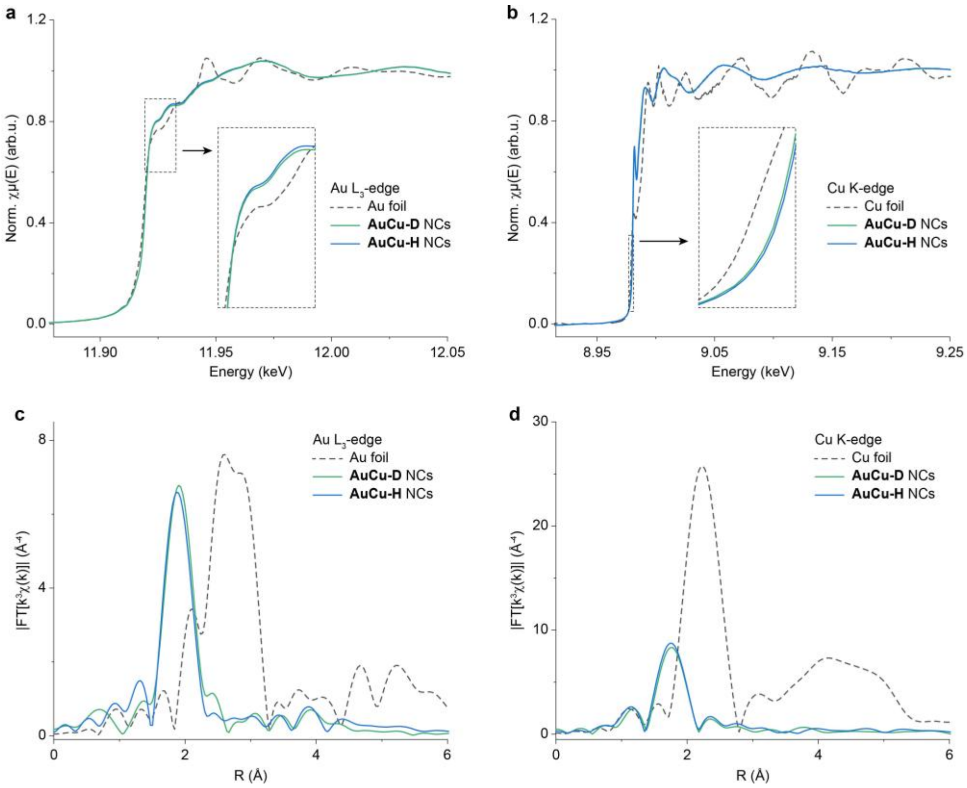

Supplementary Fig. 40. Local structure of Au and Cu atoms in AuCu-D and AuCu-H NCs. **a** Au L_3-edge XANES spectra of Au foil, AuCu-D, and AuCu-H NCs. Inset shows the magnified XANES spectra in the energy range of 11.919-11.933 keV. **b** Cu K-edge XANES spectra of Cu foil, AuCu-D, and AuCu-H NCs. Inset shows the magnified XANES spectra in the energy range of 8.977-8.980 keV. **c** Au L_3-edge FT-EXAFS spectra of Au foil, AuCu-D, and AuCu-H NCs, shown in k^3-weighted *R*-space. **d** Cu K-edge FT-EXAFS spectra of Cu foil, AuCu-D, and AuCu-H NCs, shown in k^3-weighted *R*-space.

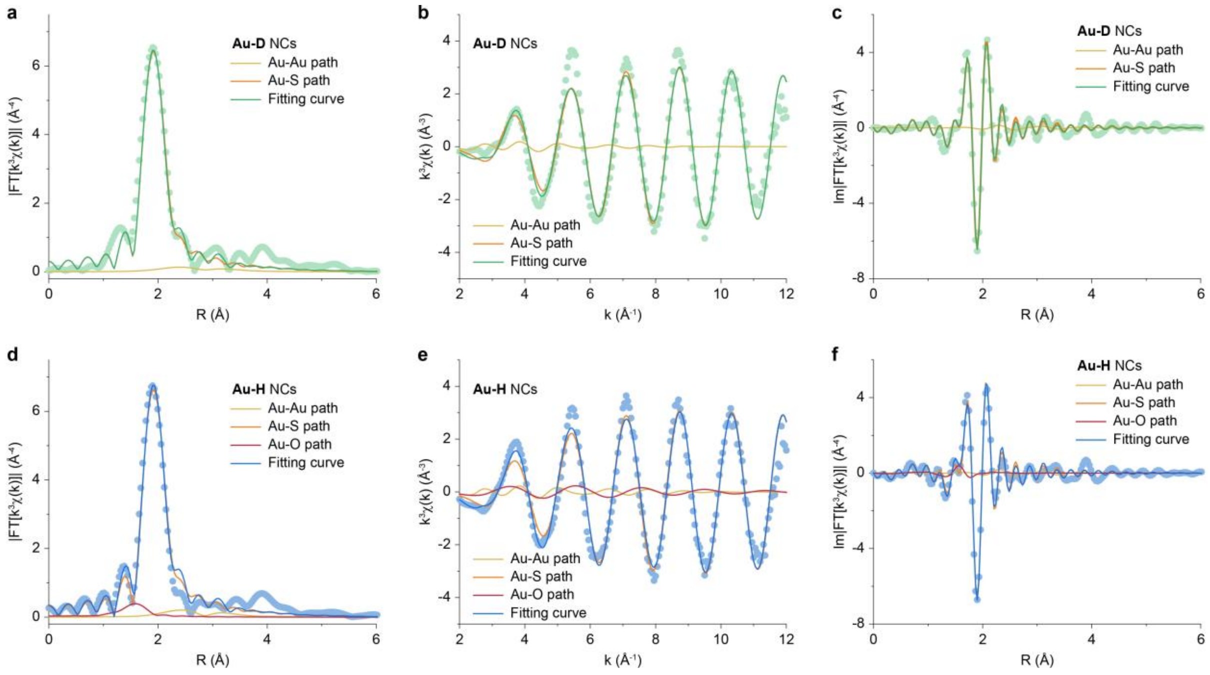

Supplementary Fig. 41. Au L_3-edge EXAFS data analysis of Au-D and Au-H NCs. a, **d** The experimental data and corresponding best fits of Au L_3-edge FT-EXAFS of Au-D and Au-H NCs, respectively, shown in k^3-weighted *R*-space. **b**, **e** The experimental data and corresponding best fits of Au L_3-edge EXAFS of Au-D and Au-H NCs, respectively, shown in k^3-weighted *k*-space. **c**, **f** The experimental data and corresponding best fits of Au L_3-edge FT-EXAFS of Au-D and Au-H NCs, respectively, shown in k^3-weighted *R*-space (imaginary component).

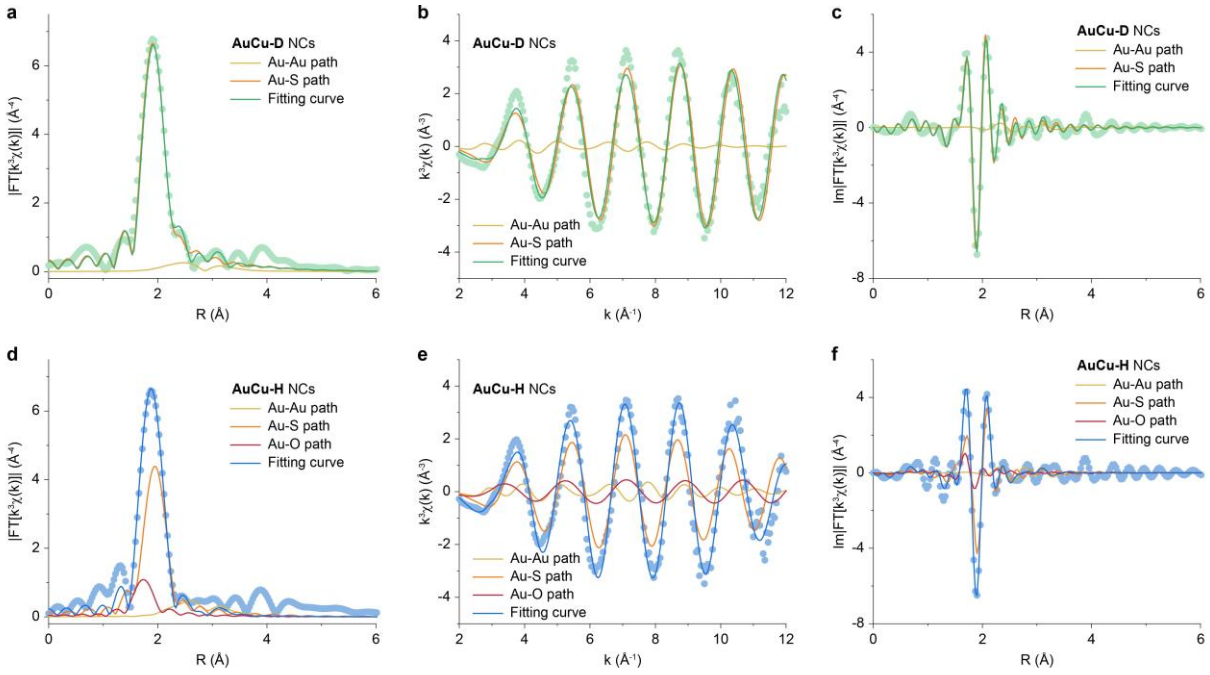

Supplementary Fig. 42. Au L_3-edge EXAFS data analysis of AuCu-D and AuCu-H NCs. **a**, **d** The experimental data and corresponding best fits of Au L_3-edge FT-EXAFS of AuCu-D and AuCu-H NCs, respectively, shown in k^3-weighted *R*-space. **b**, **e** The experimental data and corresponding best fits of Au L_3-edge EXAFS of AuCu-D and AuCu-H NCs, respectively, shown in k^3-weighted *k*-space. **c**, **f** The experimental data and corresponding best fits of Au L_3-edge FT-EXAFS of AuCu-D and AuCu-H NCs, respectively, shown in k^3-weighted *R*-space (imaginary component).

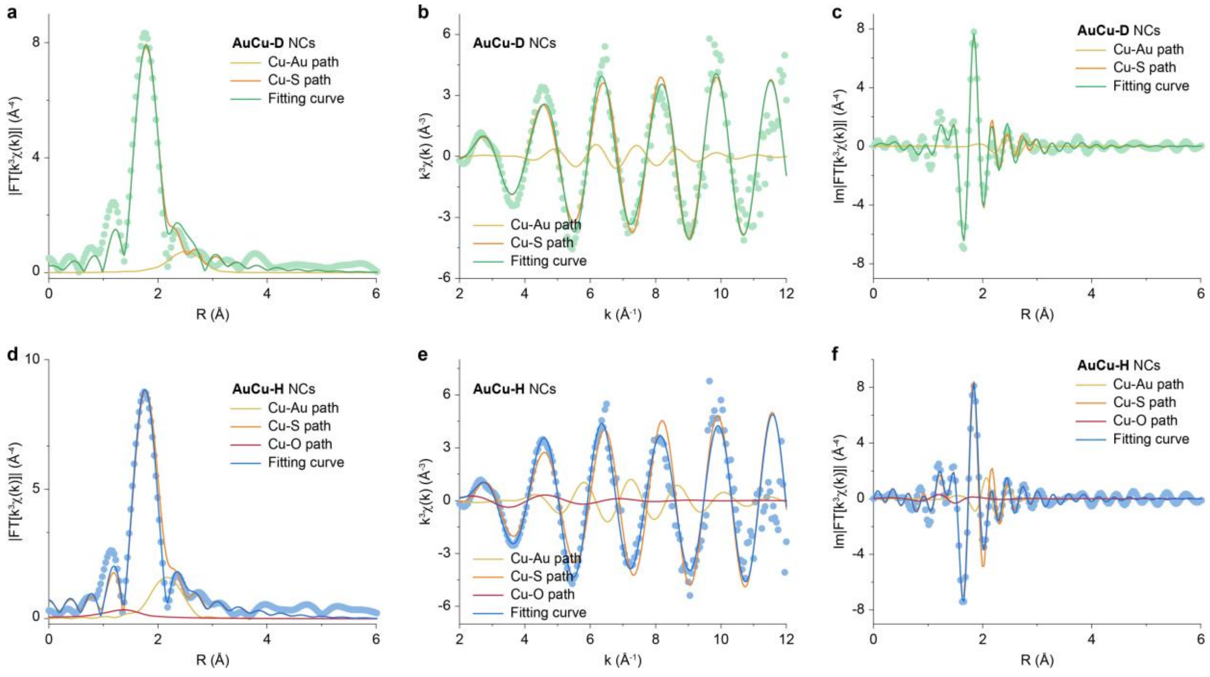

Supplementary Fig. 43. Cu K-edge EXAFS data analysis of AuCu-D and AuCu-H NCs. a, **d** The experimental data and corresponding best fits of Cu K-edge FT-EXAFS of AuCu-D and AuCu-H NCs, respectively, shown in k^3-weighted *R*-space. **b**, **e** The experimental data and corresponding best fits of Cu K-edge EXAFS of AuCu-D and AuCu-H NCs, respectively, shown in k^3-weighted *k*-space. **c**, **f** The experimental data and corresponding best fits of Cu K-edge FT-EXAFS of AuCu-D and AuCu-H NCs, respectively, shown in k^3-weighted *R*-space (imaginary component).

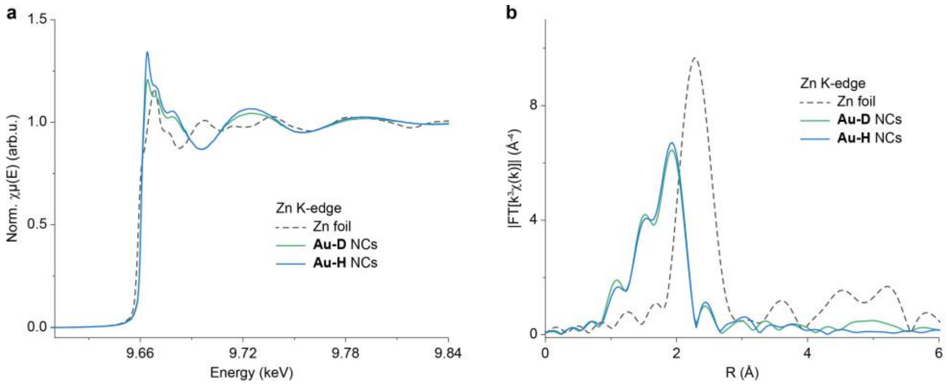

Supplementary Fig. 44. Local structure of Zn atoms in Au-D and Au-H NCs. a Zn K-edge XANES spectra of Zn foil, Au-D, and Au-H NCs. **b** Zn K-edge FT-EXAFS spectra of Zn foil, Au-D, and Au-H NCs, shown in k^3-weighted *R*-space.

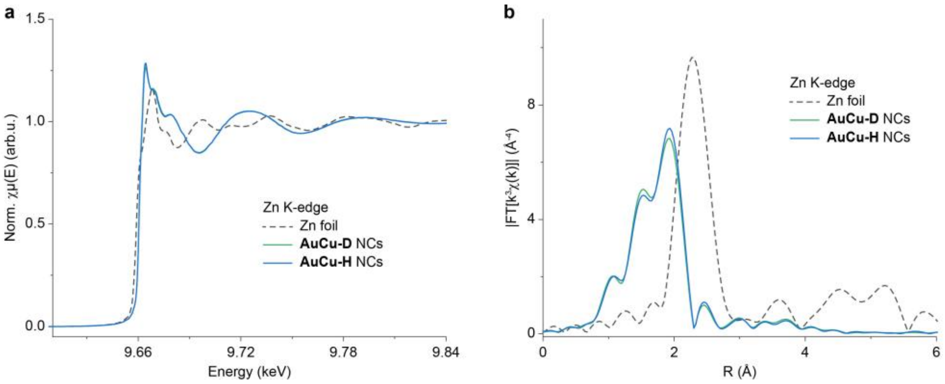

Supplementary Fig. 45. Local structure of Zn atoms in AuCu-D and AuCu-H NCs. a Zn K-edge XANES spectra of Zn foil, AuCu-D, and AuCu-H NCs. **b** Zn K-edge FT-EXAFS spectra of Zn foil, AuCu-D, and AuCu-H NCs, shown in k^3-weighted *R*-space.

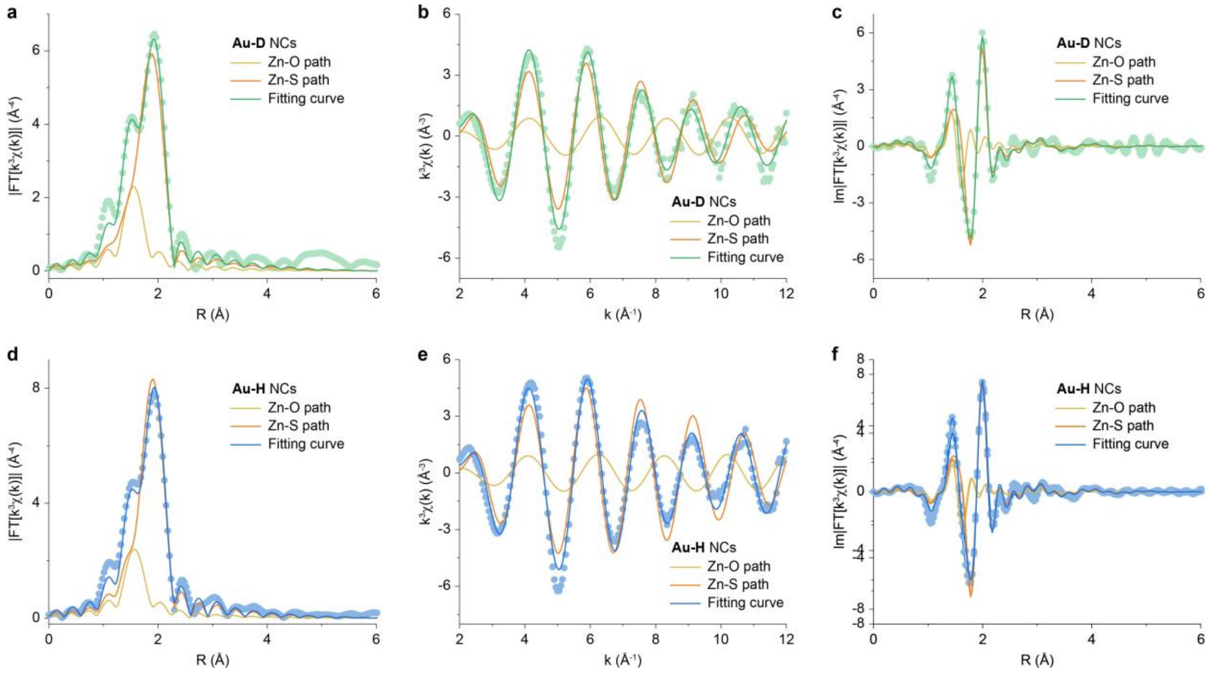

Supplementary Fig. 46. Zn K-edge EXAFS data analysis of Au-D and Au-H NCs. a, **d** The experimental data and corresponding best fits of Zn K-edge FT-EXAFS of Au-D and Au-H NCs, respectively, shown in k^3-weighted *R*-space. **b**, **e** The experimental data and corresponding best fits of Zn K-edge EXAFS of Au-D and Au-H NCs, respectively, shown in k^3-weighted *k*-space. **c**, **f** The experimental data and corresponding best fits of Zn K-edge FT-EXAFS of Au-D and Au-H NCs, respectively, shown in k^3-weighted *R*-space (imaginary component).

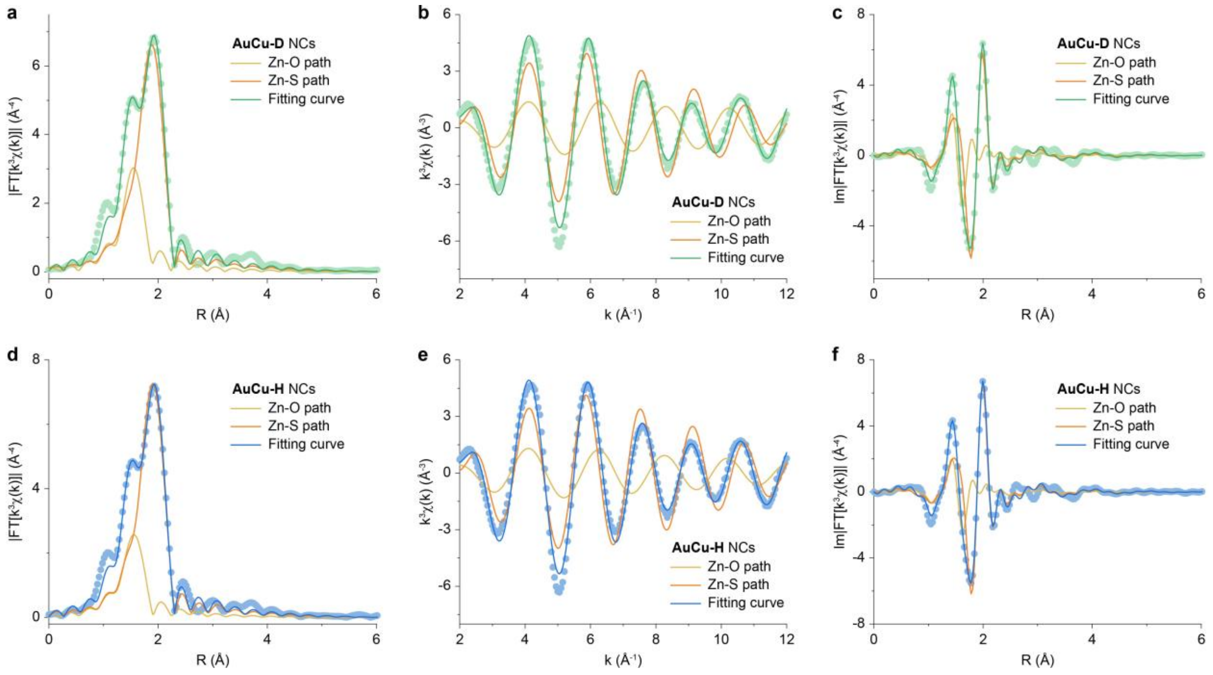

Supplementary Fig. 47. Zn K-edge EXAFS data analysis of AuCu-D and AuCu-H NCs. a, **d** The experimental data and corresponding best fits of Zn K-edge FT-EXAFS of AuCu-D and AuCu-H NCs, respectively, shown in k^3-weighted *R*-space. **b**, **e** The experimental data and corresponding best fits of Zn K-edge EXAFS of AuCu-D and AuCu-H NCs, respectively, shown in k^3-weighted *k*-space. **c**, **f** The experimental data and corresponding best fits of Zn K-edge FT-EXAFS of AuCu-D and AuCu-H NCs, respectively, shown in k^3-weighted *R*-space (imaginary component).

Supplementary Tables

Supplementary Table 1. The full width at half maximum (FWHM) and Commission Internationale de L'Eclairage (CIE) chromaticity coordinate of AuAg-D, AuAg-D aged in 2%-56% RH environment, and AuAg-H NCs aged in different RH conditions.

Sample	AuAg-D	AuAg-D in 2% RH	AuAg-D in 10% RH	AuAg-D in 15% RH	AuAg-D in 20% RH
FWHM (meV)	223.5	220.2	212.1	208.4	210.4
CIE (*x*, *y*)	(0.23, 0.67)	(0.12, 0.56)	(0.10, 0.52)	(0.09, 0.47)	(0.09, 0.42)
Sample	AuAg-D in 30% RH	AuAg-D in 40% RH	AuAg-D in 50% RH	AuAg-D in 56% RH	AuAg-S
FWHM (meV)	205.6	200.1	183.9	167.6	130.5
CIE (*x*, *y*)	(0.08, 0.38)	(0.08, 0.33)	(0.09, 0.27)	(0.10, 0.18)	(0.10, 0.17)

Supplementary Table 2. PL band deconvolutions of AuAg-D, AuAg-D aged in 2%-56% RH environment, and AuAg-S NCs. The fractions were calculated based on the integrated PL area.

Sample	PL peak (eV)	PL I peak (eV)	PL I fraction (%)	PL II peak (eV)	PL II fraction (%)	R^2 factor
AuAg-D	2.33	2.55	5.6	2.38	94.4	0.998
AuAg-D in 2% RH	2.41	2.55	27.4	2.40	72.6	0.998
AuAg-D in 10% RH	2.44	2.55	29.7	2.42	70.3	0.999
AuAg-D in 15% RH	2.47	2.55	36.1	2.42	63.9	0.999
AuAg-D in 20% RH	2.48	2.55	53.8	2.42	46.2	0.998
AuAg-D in 30% RH	2.50	2.55	68.5	2.42	31.5	0.998
AuAg-D in 40% RH	2.52	2.55	77.9	2.42	22.1	0.998
AuAg-D in 50% RH	2.54	2.55	87.6	2.42	12.4	0.999
AuAg-H in 56% RH (AuAg-H)	2.55	2.55	93.2	2.42	6.8	0.999
AuAg-S	2.58	2.58	100.0	-	-	1.000

Supplementary Table 3. Fitting results of PL lifetimes of AuAg-D, AuAg-H, and AuAg-S NCs at room temperature.

Sample	Exc. and em. wavelength (nm)	τ_1 (ns)	A_1 (fraction)	τ_2 (ns)	A_2 (fraction)	τ_3 (ns)	A_3 (fraction)	τ_{ave} (ns)	χ^2
AuAg-D	370 nm exc. 536 nm em.	16.9	56%	302.4	21%	4241.4	23%	1048.5	1.1105
AuAg-H	370 nm exc. 482 nm em.	29.5	73%	72.8	27%	-	-	41.6	1.1439
AuAg-S	370 nm exc. 480 nm em.	-	-	69.5	100%	-	-	69.5	1.0975

Supplementary Table 4. Temperature-dependent PL lifetimes of AuAg-D NCs upon 375 nm laser excitation and monitored at 536 nm.

Temperature (K)	τ_1 (ns)	A_1 (fraction)	τ_2 (ns)	A_2 (fraction)	τ_3 (ns)	A_3 (fraction)	τ_{ave} (ns)
290	22.7	57.5%	225.4	16.5%	3900.8	26.0%	1063.1
270	23.4	65.9%	156.2	12.9%	2100.7	21.2%	480.7
250	23.6	63.4%	136.8	15.3%	2580.2	21.3%	583.9
230	25.8	64.8%	163.7	17.9%	2231.5	17.3%	431.9
210	30.1	67.4%	260.1	14.5%	2935.4	18.1%	589.0
190	31.0	64.6%	175.3	19.8%	2057.4	15.6%	375.1
170	35.5	63.2%	162.0	20.5%	1707.1	16.3%	334.0
150	36.8	56.0%	141.9	30.0%	1459.2	14.0%	268.1
130	55.7	62.1%	200.0	24.5%	2032.9	13.4%	355.8
110	72.8	53.4%	268.4	33.7%	2163.3	12.9%	408.7
90	111.2	48.1%	408.0	39.5%	2766.3	12.4%	556.9
70	160.2	34.6%	539.6	49.8%	2555.6	15.6%	721.2
50	254.5	29.3%	836.3	54.6%	3813.2	16.1%	1144.9
30	293.0	19.2%	906.6	56.6%	3036.7	24.2%	1303.2
10	-	-	582.5	47.1%	2095.1	52.9%	1383.1

Supplementary Table 5. Temperature-dependent PL lifetimes of AuAg-H NCs upon 375 nm laser excitation and monitored at 482 nm.

Temperature (K)	τ_1 (ns)	A_1 (fraction)	τ_2 (ns)	A_2 (fraction)	τ_{ave} (ns)
290	50.1	73.4%	157.2	26.6%	61.5
270	56.9	76.4%	221.1	23.6%	69.4
250	56.0	66.6%	141.6	33.4%	70.9
230	63.4	72.4%	174.5	27.6%	75.4
210	68.4	73.1%	207.0	26.9%	83.6
190	79.1	74.7%	225.6	25.3%	92.8
170	89.1	71.2%	247.1	28.8%	109.3
150	112.5	74.2%	359.0	25.8%	136.8
130	140.1	70.3%	416.8	29.7%	177.9
110	191.8	66.9%	388.3	33.1%	252.9
90	292.8	63.4%	852.4	36.6%	408.1
70	480.4	51.4%	1150.6	48.6%	698.8
50	868.8	53.9%	1883.4	46.1%	1174.2
30	1231.8	49.5%	2446.2	50.5%	1651.2
10	1463.7	41.9%	2810.4	58.1%	2030.8

Supplementary Table 6. Calculation of room-temperature radiative decay rate (k_r) and nonradiative decay rate (k_{nr}) of AuAg-D, AuAg-H, and AuAg-S NCs.

Sample	PLQY<br>365 nm exc.	PLQY<br>Near band gap exc.	τ_{ave} (ns)	k_r ($\times 10^4$ s^{-1})	k_{nr} ($\times 10^5$ s^{-1})
AuAg-D	6.6%	5.3%<br>(435 nm exc.)	1048.5	5.1	9.0
AuAg-H	76.7%	79.5%<br>(410 nm exc.)	41.6	1911.1	49.3
AuAg-S	85.1%	91.6%<br>(410 nm exc.)	69.5	1318.0	12.1

Supplementary Table 7. TRPL fitting results of AuAg-D and AuAg-H NCs upon 375 nm laser excitation.

Sample	monitoring wavelength (nm)	τ_1 (ns)	A_1 (fraction)	τ_2 (ns)	A_2 (fraction)	τ_3 (ns)	A_3 (fraction)	τ_{ave} (ns)	χ^2
AuAg-D	480	13.4	56.1%	288.7	19.2%	4901.0	24.7%	1273.5	1.1105
	490	13.0	53.2%	274.4	18.8%	4808.7	22.2%	1126.0	1.1439
	500	13.1	56.6%	236.3	17.2%	4628.9	24.2%	1168.3	1.1160
	510	13.8	58.6%	246.0	17.7%	4635.4	23.7%	1150.2	1.2155
	520	14.8	58.5%	268.0	19.6%	4701.7	21.9%	1090.9	1.2037
	530	15.8	55.4%	275.0	20.9%	4675.9	23.7%	1174.4	1.2568
	540	17.3	49.3%	287.5	22.9%	4694.5	27.7%	1374.7	1.1943
	550	19.9	42.6%	321.9	26.0%	4762.9	31.4%	1587.7	1.2994
	560	24.7	37.3%	353.8	28.3%	4832.5	34.4%	1771.7	1.2106
	570	32.1	34.2%	380.8	29.8%	4872.5	36.0%	1878.6	1.3074
	580	48.6	34.3%	234.1	23.9%	4574.8	47.1%	2227.4	1.2488
AuAg-H	480	29.2	78.9%	107.2	21.1%	-	-	45.7	1.2179
	490	32.2	79.5%	178.5	20.5%	-	-	62.2	1.1954
	500	36.8	77.8%	217.6	22.2%	-	-	76.9	1.2305
	510	42.3	74.5%	247.1	25.5%	-	-	94.5	1.2516
	520	47.2	70.8%	277.3	29.2%	-	-	114.4	1.3485

Supplementary Table 8. Fitting results of Au L_3-edge FT-EXAFS in k^3-weighted *R*-space.

Sample	Shell	C.N.[a]	R (Å)[b]	σ^2 ($Å^2$)[c]	ΔE_0 (eV)[d]	R-factor[e]
Au foil	Au-Au	12.0	2.86	0.0078	4.0	0.0030
Au-D	Au-S	1.6	2.30	0.0010	8.8	0.0274
	Au-Au	2.4	2.92	0.0252		
Au-H	Au-O	0.3	1.99	0.0078	9.2	0.0244
	Au-S	1.5	2.30	0.0007		
	Au-Au	2.4	2.92	0.0259		
AuAg-D	Au-S	1.5	2.31	0.0012	9.5	0.0291
	Au-Au	2.0	2.93	0.0227		
AuAg-H	Au-O	0.4	2.05	0.0002	9.9	0.0147
	Au-S	1.5	2.32	0.0012		
	Au-Au	2.0	2.93	0.0321		
AuCu-D	Au-S	1.6	2.31	0.0012	9.5	0.0273
	Au-Au	2.0	2.92	0.0199		
AuCu-H	Au-O	0.4	2.09	0.0057	9.3	0.0167
	Au-S	1.5	2.34	0.0015		
	Au-Au	2.0	2.93	0.0204		

[a]: coordination number; [b]: distance between absorber and backscatter atoms; [c]: Debye-Waller factors; [d]: inner potential correction; [e]: indicative of fitting goodness. Note that the Fourier transformation of the k^3-weighted EXAFS oscillations was performed over a range of 3.0-10.5 $Å^{-1}$. The best fits to the EXAFS signal were made in *R*-space in the interval 1.0-3.0 Å. The overall amplitude reduction factor, S_0^2, was fixed at the best-fit value of 0.81 for all NCs, which was determined from fitting the data of Au foil.

Supplementary Table 9. Fitting results of Ag K-edge FT-EXAFS in k^3-weighted *R*-space.

Sample	Shell	C.N.[a]	R (Å)[b]	σ^2 ($Å^2$)[c]	ΔE_0 (eV)[d]	R-factor[e]
Ag foil	Ag-Ag	12.0	2.86	0.0094	0.0	0.0038
AuAg-D	Ag-S	1.4	2.40	0.0035	2.3	0.0295
	Ag-Au	2.0	3.04	0.0380		
AuAg-H	Ag-O	0.5	2.15	0.0142	4.3	0.0242
	Ag-S	1.4	2.41	0.0038		
	Ag-Au	2.0	3.04	0.0355		

[a]: coordination number; [b]: distance between absorber and backscatter atoms; [c]: Debye-Waller factors; [d]: inner potential correction; [e]: indicative of fitting goodness. Note that the Fourier transformation of the k^3-weighted EXAFS oscillations was performed over a range of 3.0-10.5 $Å^{-1}$. The best fits to the EXAFS signal were made in *R*-space in the interval 1.0-3.0 Å. The overall amplitude reduction factor, S_0^2, was fixed at the best-fit value of 0.83 for all NCs, which was determined from fitting the data of Ag foil.

Supplementary Table 10. Fitting results of Cu K-edge FT-EXAFS in k^3-weighted *R*-space.

Sample	Shell	C.N.[a]	R (Å)[b]	σ^2 ($Å^2$)[c]	ΔE_0 (eV)[d]	R-factor[e]
Cu foil	Cu-Cu	12.0	2.54	0.0079	4.3	0.0115
AuCu-D	Cu-S	1.4	2.18	0.0005	2.1	0.0285
	Cu-Au	2.0	2.89	0.0179		
AuCu-H	Cu-O	0.5	1.85	0.0209	3.0	0.0129
	Cu-S	1.4	2.18	0.0006		
	Cu-Au	2.0	2.82	0.0142		

[a]: coordination number; [b]: distance between absorber and backscatter atoms; [c]: Debye-Waller factors; [d]: inner potential correction; [e]: indicative of fitting goodness. Note that the Fourier transformation of the k^3-weighted EXAFS oscillations was performed over a range of 3.0-10.5 $Å^{-1}$. The best fits to the EXAFS signal were made in *R*-space in the interval 1.0-3.0 Å. The overall amplitude reduction factor, S_0^2, was fixed at the best-fit value of 0.85 for all NCs, which was determined from fitting the data of Cu foil.

Supplementary Table 11. Fitting results of Zn K-edge FT-EXAFS in k^3-weighted *R*-space.

Sample	Shell	C.N.[a]	R (Å)[b]	σ^2 ($Å^2$)[c]	ΔE_0 (eV)[d]	R-factor[e]
Zn foil	Zn-Zn	6.0	2.63	0.0111	1.6	0.0248
Au-D	Zn-O	0.5	1.94	0.0003	2.6	0.0161
	Zn-S	2.7	2.32	0.0081		
Au-H	Zn-O	0.5	1.96	0.0002	3.2	0.0177
	Zn-S	2.7	2.32	0.0060		
AuAg-D	Zn-O	0.8	1.94	0.0017	2.3	0.0125
	Zn-S	2.9	2.31	0.0089		
AuAg-H	Zn-O	0.8	1.96	0.0018	3.0	0.0127
	Zn-S	2.9	2.32	0.0077		
AuCu-D	Zn-O	0.9	1.95	0.0022	2.5	0.0124
	Zn-S	2.9	2.31	0.0080		
AuCu-H	Zn-O	0.9	1.95	0.0027	3.0	0.0121
	Zn-S	2.9	2.32	0.0076		

[a]: coordination number; [b]: distance between absorber and backscatter atoms; [c]: Debye-Waller factors; [d]: inner potential correction; [e]: indicative of fitting goodness. Note that the Fourier transformation of the k^3-weighted EXAFS oscillations was performed over a range of 3.0-10.5 $Å^{-1}$. The best fits to the EXAFS signal were made in *R*-space in the interval 1.0-3.0 Å. The overall amplitude reduction factor, S_0^2, was fixed at the best-fit value of 0.98 for all NCs, which was determined from fitting the data of Zn foil.

Supplementary References

1. Liu, K. et al., Vibrational strong coupling between surface phonon polaritons and organic molecules via single quartz micropillars. *Adv. Mater.* **34**, 2109088 (2022).
2. Chevrier, D. M. et al., Interactions between ultrastable $Na_4Ag_{44}(SR)_{30}$ nanoclusters and coordinating solvents: Uncovering the atomic-scale mechanism. *ACS Nano* **14**, 8433–8441 (2020).
3. Shanthi, P. M. et al., Sulfonic acid based complex framework materials (CFM): nanostructured polysulfide immobilization systems for rechargeable lithium–sulfur battery. *J. Electrochem. Soc.* **166**, A1827 (2019).
