# Supplementary material for: Lighting up metal nanoclusters by the H2O-dictated electron relaxation dynamics
Source: Nat Commun. 2025 Mar 7;16:2295. doi: 10.1038/s41467-025-57554-7 (PMC11889173; doi:10.1038/s41467-025-57554-7)
Supplement: Supplementary file 2 — Description of Additional Supplementary Files [file 41467_2025_57554_MOESM2_ESM.pdf]

Description of Additional Supplementary Files:

Supplementary Data 1: The calculated atomic coordinates of AuAg-D NCs.

Supplementary Data 2: The calculated atomic coordinates of AuAg-H-H_2O@Au NCs.

Supplementary Data 3: The calculated atomic coordinates of AuAg-H-H_2O@Ag NCs.

Supplementary Movie 1: Spontaneous water adsorption of AuAg-D NCs in 56% RH air.

Supplementary Movie 2: Water desorption of AuAg-H NCs by pumping vacuum.
